# Molecular docking studies and molecular dynamic simulation analysis: To identify novel ATP-competitive inhibition of Glycogen synthase kinase-3β for Alzheimer’s disease

**DOI:** 10.12688/f1000research.145391.1

**Published:** 2024-07-08

**Authors:** Suggala Ramya Shri, Yogendra Nayak, Sreedhara Ranganath Pai

**Affiliations:** 1 Department of Pharmacology, Manipal College of Pharmaceutical Sciences, Manipal Academy of Higher Education, Manipal, Karnataka, 576104, India

**Keywords:** Alzheimer’s disease, GSK-3β, ATP-competitive study, Protein preparation, Ligand Preparation, Qikprop, Glide module, Prime MM-GBSA, Molecular dynamic simulation.

## Abstract

**Background:**

The discovery of an ideal and effective therapy is urgently required for the treatment of Alzheimer’s disease. The main pathological hallmarks of Alzheimer’s disease that appear before the clinical symptoms are neurofibrillary tangles, amyloid plaques, brain inflammation, and neuronal atrophy throughout the cerebral cortex and hippocampus. GSK-3β (Glycogen Synthase Kinase-3β) is regarded as the most important and promising target for therapeutic use because GSK-3β expression levels increase with age and are the most abundant and hyperactive in the brains of patients with Alzheimer’s disease.

**Methods:**

We used Maestro, which is Schrodinger, for our computational simulation studies. In the present work, we have used different modules that were used in previous studies with a little modification, the modules such as Protein Preparation with the help of Protein Preparation Wizard, Ligand Preparation with the help of LigPrep, for ADME (Absorption, Distribution, Metabolism and Excretion) prediction we used Qikprop, Docking studies we used Glide module, Binding energy prediction we used Prime and Molecular dynamic simulation studies by Desmond

**Results:**

Our focus is mainly on an *in-silico* approach, focusing on library generation; we first drew an imidazo [1,5-a]pyridine-3-carboxamide (IMID 2) scaffold structure at Enamine and subjected it to a substructure search to target the receptor grid region (ATP-competitive site) of 6Y9R. They were then subjected to various screening processes. Finally, we selected nine compounds and subjected them to molecular dynamic simulation studies.

**Conclusions:**

Nine compounds showed good results with the most stable interactions. Further experiments and studies are required to confirm these results.

## Introduction

One of the greatest threats to public health is neurodegenerative diseases because there is no exact therapy. Therefore, the discovery of an ideal and effective therapy is urgently needed for the treatment of Alzheimer’s disease. ^1^ Only five to seven cases are due to genetic mutations, and the remaining cases are due to environmental factors and sporadic mutations. The main pathological hallmarks of Alzheimer’s disease that appear before the clinical symptoms are neurofibrillary tangles, amyloid plaques, brain inflammation, and neuronal atrophy throughout the cerebral cortex and hippocampus. ^2^ Memory loss can be elicited in Alzheimer’s patients, such as episodic short-term memory impairment followed by a lack of motivation, disorganization, and impairment in solving problems, judgment, and executive functioning. In early stage impairments, such as visuospatial skills, neuropsychiatric symptoms are most common in mild and late-stage Alzheimer’s disease. ^3^ The heritability of AD is estimated to be between 60% and 80%, and these components allow for the identification and determination of pathophysiological processes, diagnostic markers, biological targets, and new treatment targets through genomics translational studies. ^4^ Glycogen synthase kinase-3 (GSK-3) is a member of the protein kinase family and is widely expressed in tissues. GSK-3 is a Serine/Threonine kinase that transfers a phosphate group to either the serine or threonine residue of its substrate target. ^5^ GSK-3β is regarded as the most important and promising therapeutic target. The etiology and pathogenesis of AD are not completely understood. However, available treatments have failed to show novel approaches and effectiveness, and the efficacy of drugs varies from one person to another. ^6^ GSK-3β expression levels increase with age and are most abundant and hyperactive in the brains of patients with AD. Hence, dysregulation of GSK-3β automatically affects amyloid beta plaques, which have been previously shown in in vitro and in vivo Alzheimer’s disease models. ^7^ GSK-3 plays a very important key role in the metabolic process and regulating structural processes in adult neurons as well as in developing neurons. ^8^ In the present study, we used a molecular modeling approach.

## Methods

### Software used for this computational study

We used Maestro for our computational simulation studies; the graphical interface was Schrödinger. In the present work, we used different modules that were used in previous studies with slight modifications: Protein Preparation Wizard, Ligand Preparation with the help of LigPrep, for ADME (Absorption, Distribution, Metabolism and Excretion) prediction, we used Qikprop, Docking studies we used the Glide module, and binding energy prediction we used Prime and Molecular dynamic simulation studies by Desmond. ^9^^–^^11^ Alternatively freely available software are AutoDock 4, ArgusLab, and Gromacs,

### Selection and preparation of protein

We used PDB I.D: 6Y9R ( http://www.rcsb.org/) ^12^ of GSK-3β, which is bound to a co-crystallized ligand with a Resolution of 2.08 Å consisting of only chain A, downloaded from the protein databank site, ^10^^,^^11^ and imported this PDB I. D to the Maestro interface. This is followed by preparing the protein molecule with the help of the protein preparation wizard panel of the Schrödinger suite. Alternatively, the AutoDock 4 which is freely available software can be used. The first step for protein preparation is preprocessing in the workspace the protein structure is selected then Assigned bond orders, using the CCD database, explicit hydrogen to the structure, zero-order bonds to metals, creating disulfide bonds, and optimizing missing side chain atoms by running a Prime job, fill in missing loops by running the Prime job was selected and delete waters that are further than specified distance beyond 5.00 Å from any of the het groups including ions. This is recommended for Glide and virtual screening, but Molecular Dynamics applications should keep these water as they will help the equilibrate the solvent box faster and generate het states using Epik pH: 7.5±0.0, and clicked for preprocessing. The second step is to review and modify here when a new chain, water, or het is selected to zoom to fit the selection to the workspace, select waters and hets within 5.0 Å of selected chains, and keep the remaining residues and chains as the default setting and generate a state pH of 7.5±0.0. The third step is to refine we have selected sample water orientations in addition to other groups, and the protonation states of residues and ligands were set by using PROPKA pH: 7.5 then it simulated the exact experimental conditions and clicked to automatically optimize hydroxyl, Asn, Gln, His states using ProtAssign, removing water that is further than beyond hets 3.0 Å, including ions. This is recommended for Glide and virtual screening, but Molecular Dynamics applications should keep this water as it will help equilibrate the solvent box faster. We used the OPLS3e force field for the restrained minimization of proteins. After minimization, the glycerol and acetate ions were removed from the minimized protein.

### Ligand selection and preparation

Imidazo [1,5-a]pyridine-3-carboxamide (IMID 2) scaffolds were used for this study. This scaffold possesses fewer hydrogen bond donors and improved Central Nervous System (CNS) penetration at micromolar concentrations (μM), based on studies by Buonfiglio et al. ^13^ To generate the focus library, we first drew an imidazo[1,5-a]pyridine-3-carboxamide (IMID 2) scaffold structure at Enamine and subjected it to a substructure search. Then, we obtained enamine realdb_IMID2_Scaffold_molecules (1400 molecules), followed by ligand preparation using LigPrep in the Schrödinger suite software (Schrödinger 2021-3). ^14^ Alternatively, the AutoDock 4 which is freely available software can be used. The 3D coordinates for the compounds were generated using the LigPrep tool, and the Epik module predicted the most probable charge form for the ionization state of compounds at pH: 7.5±0.0, generated tautomers and stereoisomers, determined chiralities from the 3D structure, generated at most 32 per ligand, and finally, the minimization of the drug molecule process was performed using the force field OPLS3e. ^15^^,^^16^

### ADME prediction

After ligand preparation, all ligands were subjected to Qikprop in the Schrödinger suite software to predict the ADME profile of the compounds. QikProp predicts the drug-like properties of all selected compounds, such as molecular weight, Hydrogen Bond Donors, Hydrogen Bond Acceptor, QPlogS (predicted aqueous solubility), QPlogPo/w (predicted octanol/water partition coefficient), QPlogBB (predicted brain/blood partition coefficient), QPlogPw (predicted water/gas partition coefficient), QPlogPC16 (predicted hexadecane/gas partition coefficient), QPlogPoct (predicted octanol/gas partition coefficient), PSA (Van der Waals surface area of polar nitrogen and oxygen atoms), QPPCaco (predicted apparent Caco-2 cell permeability), and QPPMDCK (predicted apparent MDCK cell permeability). ^17^

### Molecular docking studies

Here, we used the Schrödinger suite Glide module to better understand the prediction of the protein-ligand involved in assessing the fitting of all conformations of compounds at the binding site followed by ranking and modes Schrödinger Release 2021-3. The alternative freely accessible softwares are AutoDock and Gromacs. The molecular docking study process was used for assessment before molecular docking studies, and the default settings were used for the Receptor Grid generation module. The binding site was recognized using the Receptor Grid generation module, and this binding site was specified as a box of 10 × 10 × 10 Å ^3^ centered on the centroid of the co-crystallized ligand of GSK-3β, which was taken into consideration for grid generation. ^18^ We subjected all prepared compounds to the standard precision (SP) scoring function mode. ^19^ Glide SP docking ligand sampling is in flexible mode and selected sample nitrogen inversions, sample ring conformations, and amides only penalize non-planar conformations, adding Epik state penalties to the docking score. In the output file, we selected the pose viewer file that includes the receptor, written out at most one pose per ligand, performed post-docking minimization, the number of poses per ligand to include was five, and the RMSD was computed to input ligand geometries and run the job. Finally, the results were analyzed, separated, and selected based on the VAL135 residue interaction and physicochemical properties reported in the literature. These compounds were then subjected to the prime-MMGBSA module.

### MM-GBSA binding energy

MM-GBSA (molecular mechanics-generalized born energies & surface area/accessibility continuum solvation method) was used to calculate the binding free energy and binding analysis of 523 SP docked protein-ligand complexes. MM-GBSA exhibits many energy properties for proteins, ligands, and complex structures. Prime MM-GBSA analysis is based on the solvent model of VSGB 2.0, and the OPLS3e force field is used to calculate the binding affinity of the respective protein-ligand complex. ^20^

### Molecular dynamics simulation with desmond

This model was used to precisely predict the interaction of the ligand with 6Y9R (protein), the stability of their binding under physiological conditions, and to analyze their motion at the molecular and atomic levels. The protein-ligand complex was subjected to a system built here, and different solvent models were selected; an orthorhombic box with dimensions of 10 Å × 10 Å × 10 Å was used to determine the structure geometry to have the minimum box volume, and the checked boundary box is shown in the workspace. The next step is to load the previous system builder file and enter 100ns in simulation time, 1000frames were captured, ensemble class is NPT (Normal Pressure Temperature), 300°K temperature, pressure bar is 1, and relax model system before simulation was used for molecular dynamic simulation studies. ^21^ After Molecular dynamic simulation, the analysis was done with the help of the RMSD (Root Mean Square Deviation), the RMSD of ligand, RMSD of protein. RMSD is mainly used to determine the stability of the protein-ligand complex, RMSF (Root Mean Square Fluctuations), ligand-protein contacts, and ligand interaction diagram. Alternatively, NAMD molecular simulation along with VMD software can be used which is freely available.

## Results and Discussion

When we searched for PDB in UniProt, we identified 89 GSK-3β human PDBs. out of 89 PDB entries, 8 PDB literature are yet to be published, and some PDBs with position 3-12 amino acids residual chains C and D are removed from my PDB selection list. PDBs that have chain A/B are taken into consideration, and we studied all the PDBs individually. Finally, we decided on PDB: 6Y9R. ^25^

The co-crystal structure of 2 was in complex with PDB I. D 6Y9R, which is a GSK-3β enzyme. While INDZ was located at the hinge region, the piperidine chain was exposed to the solvent towards ARG141, Pyridine formed a hydrogen bond with LYS85, nearer to the ligand several hydration sites with residues such as THR138, ASN186, GLN185, LEU132, ASP200, and IIE62 were found to be fully explained in a previous study. The INDZ core scaffold was located in the ATP-competitive binding site of GSK-3β between the N-terminal lobe and the C-terminal lobe region of the protein, and INDZ was previously developed as a novel GSK-3β inhibitor using computational tools. The N-2 of the core and VAL135 N-H group, hydrogen at position N-1 of the core with the ASP133 carbonyl group, hydrogen of the carboxamide group, and VAL135 carbonyl group are important interactions. The R1 substituent appended to the core is oriented towards ARG141 in the external solvent-accessible part of the solvent, and the R2 substituent appended to the LYS85 inner cavity proximity. The indazole N-Ha was then replaced with the C-Ha group, and the ring nitrogen atoms were shuffled, resulting in the IMID2 core. ^13^

**Figure 1.**
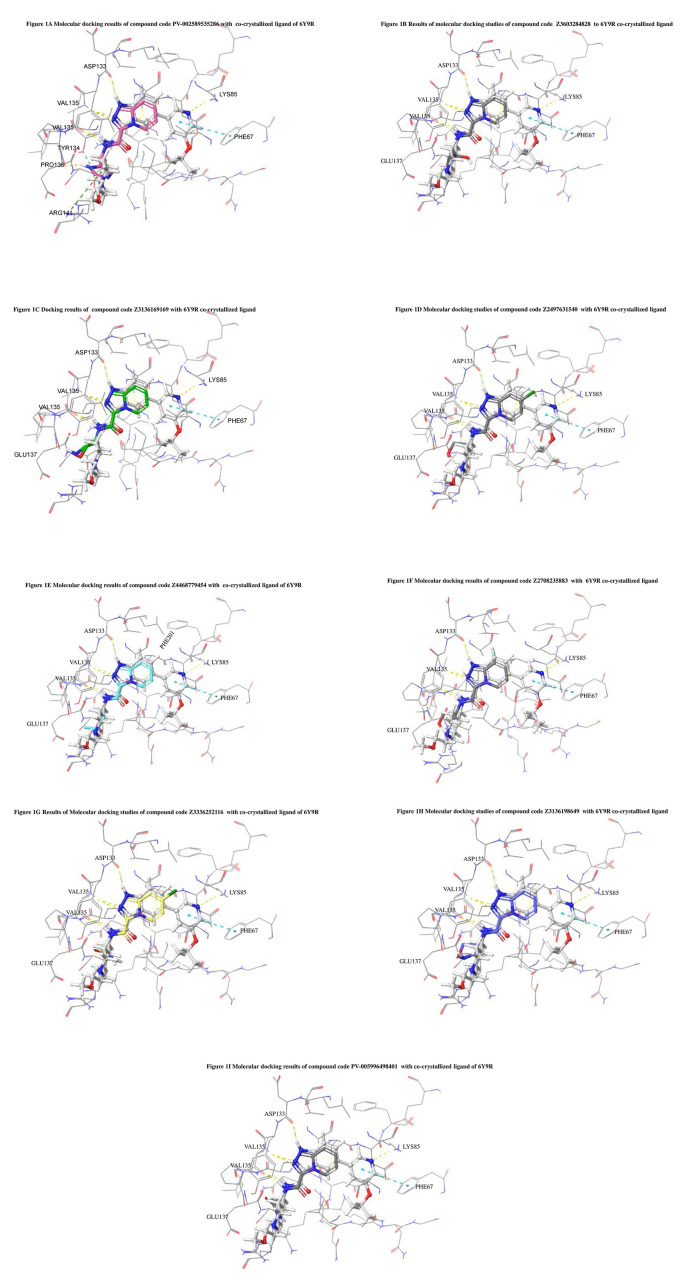
Overlap of co-crystal structure of 6Y9R and IMID2 substructures.

The docked ligands accommodated in the ATP-binding site are similar to the co-crystal structure of the 6Y9R protein. The overlap of the co-crystal structures of the 6Y9R and IMID2 substructures is shown in Figure 1 ( Figure 1A to 1I).

The docking of ligands to a particular receptor grid generated a region of the protein 6Y9R. In this study, SP docking was used. After docking, the compounds were separated and selected based on the VAL135 residue interaction in the literature. Therefore, based on the VAL135 N-H group {the H group of the VAL135 protein should show direct interaction with the nitrogen group of the ligand}. Through this process, we finalized 523 molecules of the IMID2 scaffold. Compounds with good SP-scores are listed in Table 1.

**Table 1.** Intermolecular interactions with amino acids and docking score of the top 9 selected compounds.

Name	Docking score	Structure	Bonded interaction	Non-bonded interaction
Z3336252116	-8.535	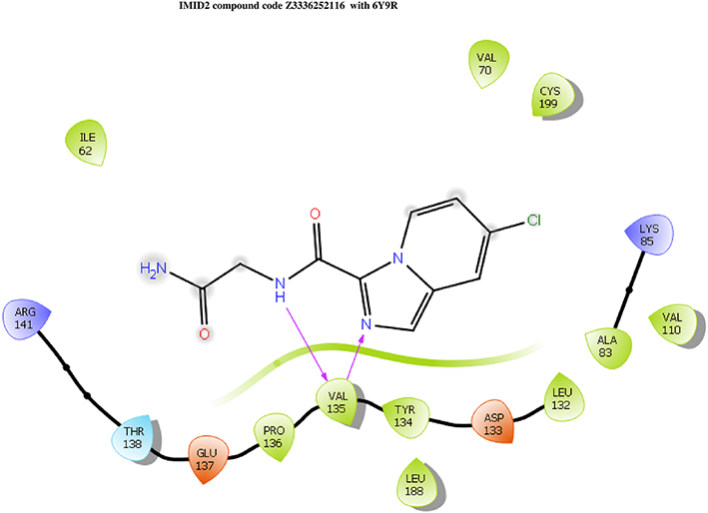	H-bond: VAL135,	ILE62, ARG141, THR138, GLU137, PRO136, TYR134, LEU188, ASP133, LEU132, ALA83, VAL110, LYS85, CYS199, VAL70
Z3136198649	-8.285	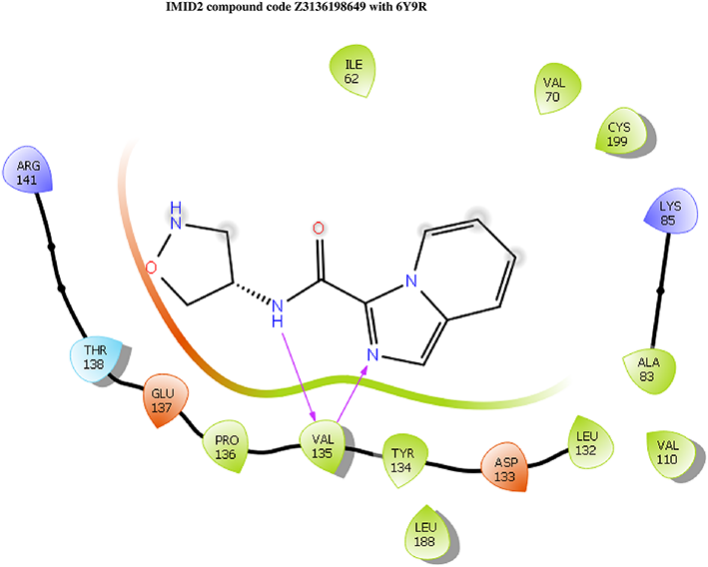	H-bond: VAL135	ILE62, ARG141, THR138, GLU137, PRO136, TYR134, LEU188, ASP133, LEU132, ALA83, VAL110, LYS85, CYS199, VAL70
PV-005996498401	-8.132	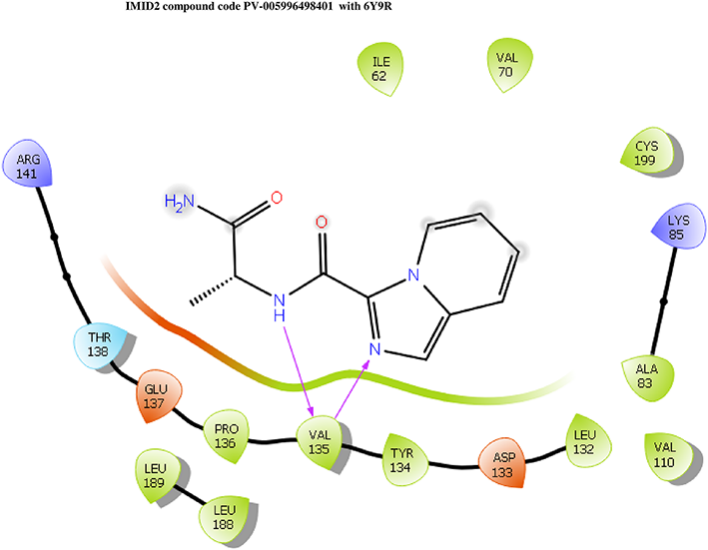	H-bond: VAL135	ARG141, THR138, GLU137, PRO136, LEU189, LEU188, TYR134, ASP133, LEU132, VAL110, ALA83, LYS85, CYS199, VAL70, ILE62
PV-0025895352867	-7.541	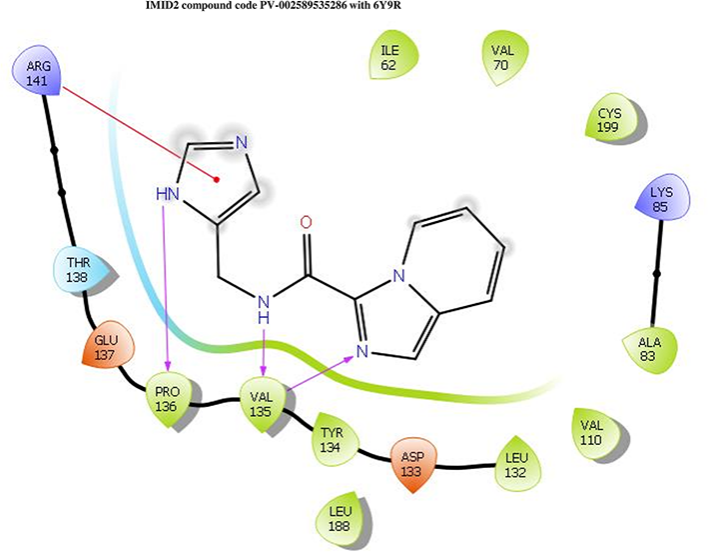	H-bond: VAL135, PRO136 PI-cation: ARG141	VAL110, ALA83, LYS85, CYS199, VAL70, ILE62, LEU132, ASP133, TYR134, LEU188, GLU137, THR138
Z3603284828	-7.765	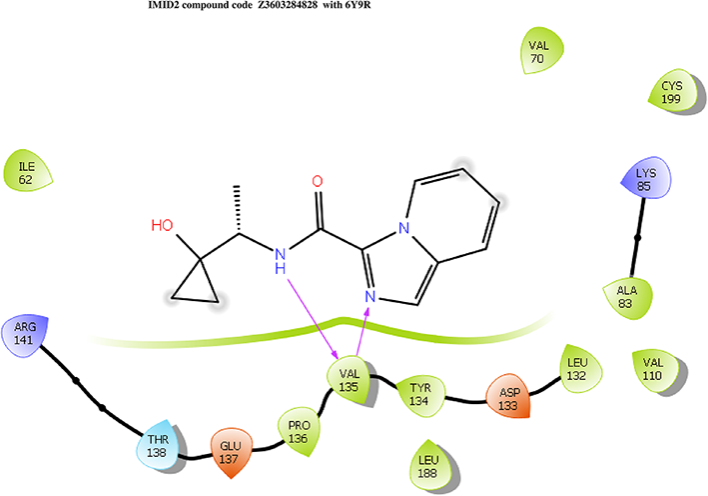	H-bond: VAL135	ILE62, ARG141, THR138, GLU137, PRO136, TYR134, LEU188, ASP133, LEU132, ALA83, VAL110, LYS85, CYS199, VAL70
Z3136169169	-8.268	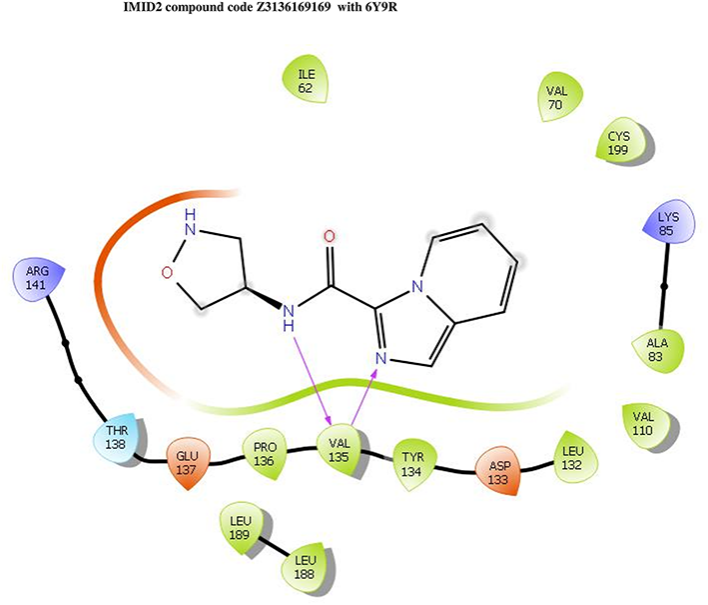	H-bond: VAL135	ARG141, THR138, GLU137, PRO136, LEU189, LEU188, TYR134, ASP133, LEU132, VAL110, ALA83, LYS85, CYS199, VAL70, ILE62
Z2497631540	-7.967	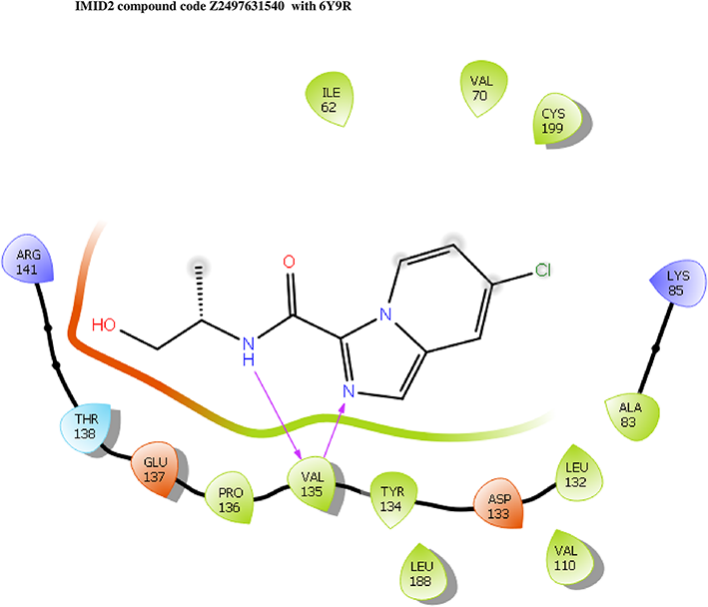	H-bond: VAL135	ARG141, THR138, GLU137, PRO136, TYR134, LEU188, ASP133, LEU132, VAL110, CYS199, ALA83, LYS85, VAL70, ILE62
Z4468779454	-7.306	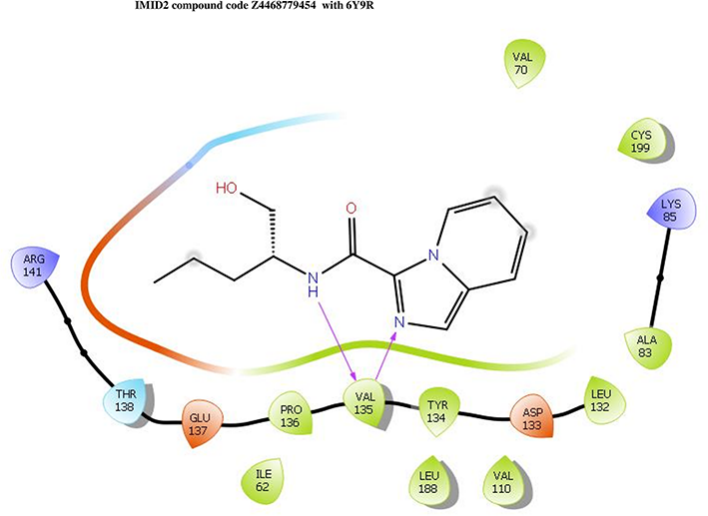	H-bond: VAL135	ARG141, THR138, GLU137, PRO136, TYR134, LEU188, ASP133, LEU132, VAL110, CYS199, ALA83, LYS85, VAL70, ILE62
Z2708235883	-7.961	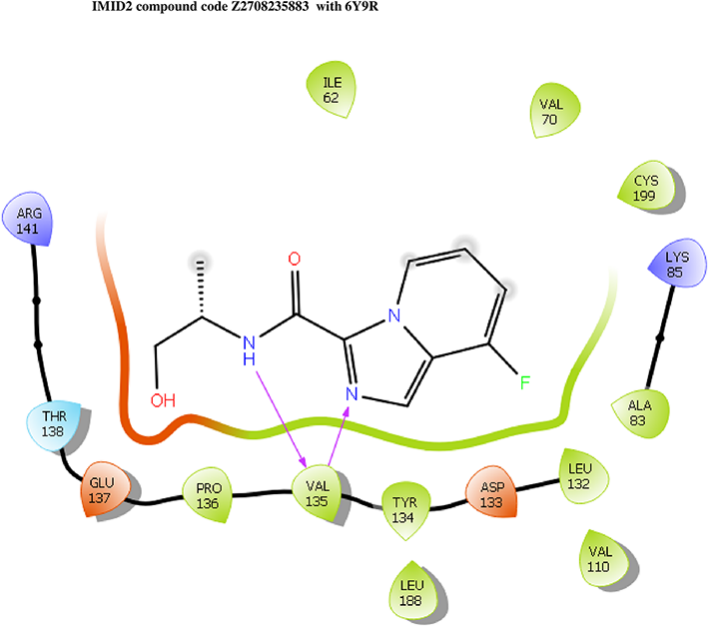	H-bond: VAL135	ARG141, THR138, GLU137, PRO136, TYR134, LEU188, ASP133, LEU132, VAL110, CYS199, ALA83, LYS85, VAL70, ILE62

### ADME prediction

The Absorption, Distribution, Metabolism, and Excretion (ADME) or drug-likeness parameters are the most important and helpful parameters for screening compounds after SP docking studies during the drug discovery process. ^22^ Generally, for CNS drugs, the physicochemical properties range from molecular weight 100 Da to 450 Da, hydrogen bond acceptors range from 0 to 5, hydrogen bond donor values range from 0 to 3, and the topological polar surface area value is ≤76 Å2. ^23^ The results of this study are listed in Table 2. Whereas the previous ADME properties of 13 trial compounds with an IMID2 core scaffold molecular weight of 393.48, the hydrogen bond donor was 1. In the14 trial compound of the IMID2 core scaffold, the molecular weight was 451.56, and the hydrogen bond donors were 1. In the 15 trial compounds of the IMID2 core scaffold, the molecular weight was 336.39, and the hydrogen bond donors were 1. In 16 trials of the IMID2 core scaffold, the molecular weight was 394.47, and the number of hydrogen bond donors was 1. ^13^

**Table 2.** MM-GBSA score and Qikprop ADME prediction results of the top-9 selected compounds.

Name	MM-GBSA score	Molecular weight	Hydrogen Bond Donor	Hydrogen Bond Acceptor	Polar Surface Area
Z3336252116	-36.94	252.660	2	3	105.021
Z3136198649	-40.67	232.243	2	4	81.022
PV-005996498401	-33.07	232.243	2	3	101.470
PV-002589535286	-37.94	241.252	2	5.5	78.034
Z3603284828	-40.91	245.282	2	3	72.320
Z3136169169	-40.53	232.243	2	4	81.001
Z2497631540	-42.12	253.688	2	5.7	73.124
Z4468779454	-46.04	247.296	2	5.7	70.083
Z2708235883	-39.16	237.233	2	5.7	73.152

### Molecular dynamic simulation analysis

Compounds were selected based on the screening process described above. Only short-listed compounds were subjected to desmonds for molecular dynamic simulations. These studies provide information related to the stability and flexibility of the molecular docking complexes. We performed this study using the MM-GBSA. This study provides information related to protein-ligand interactions, RMSD variation can be assessed, and RMSF fluctuations can be assessed for protein-ligand complexes. The complex was regarded as stable if it fell within the 3 Å range. These are the compounds

**Figure 2.**
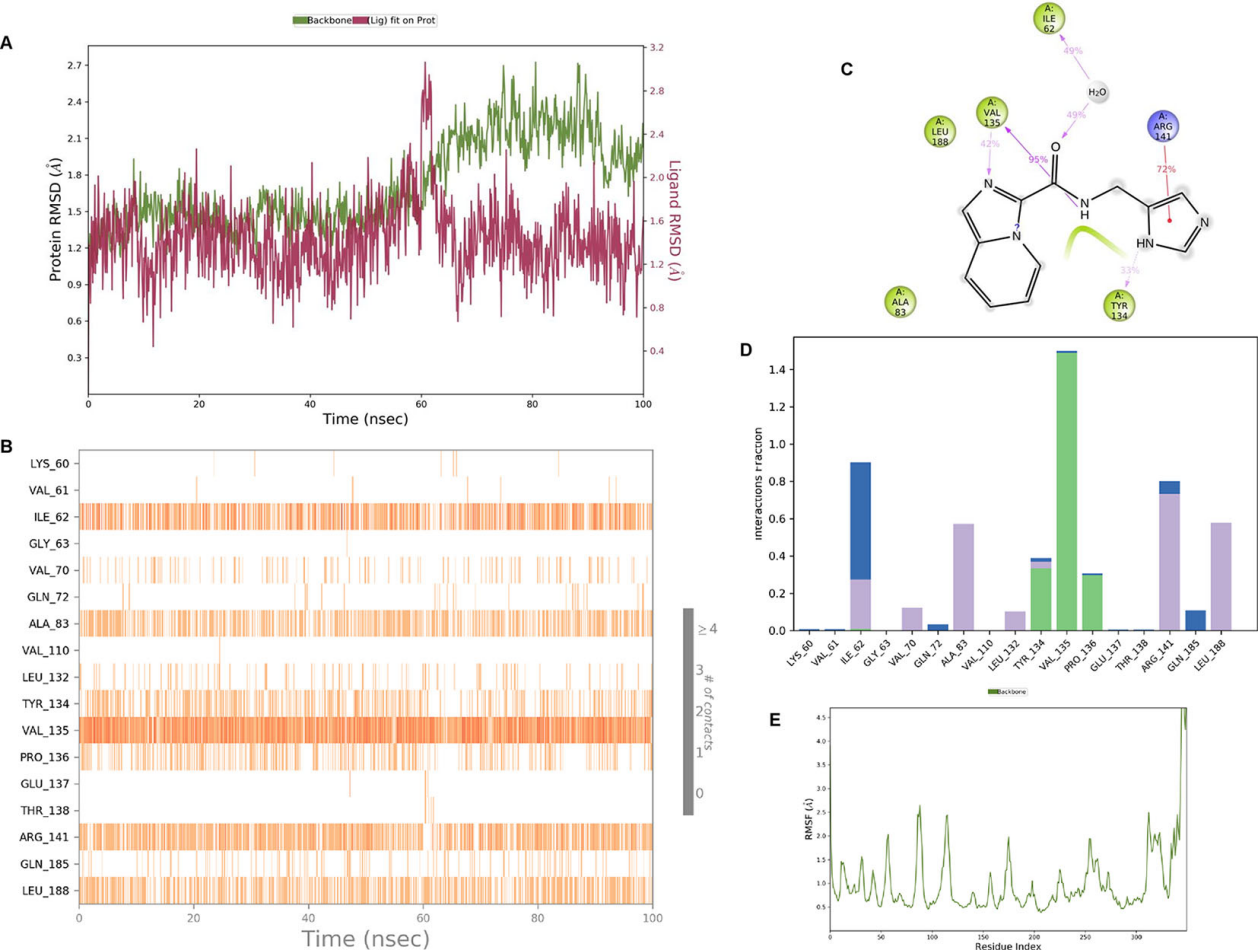
Pictorial representation of 6Y9R interactions with the IMID2 compound code PV-002589535286 monitored during the course of MD Simulation trajectory (A) Protein-Ligands RMSD; (B) Protein-Ligand Contacts; (C) Ligand-Protein Contacts; (D) Protein-Ligand Contacts described as histogram; (E) Protein RMSF.

**IMID2 PV-002589535286:** During MD simulations, the ligand-protein complex showed hydrogen-bonded interactions. The protein backbone and ligand structure exhibited higher RMSD fluctuations over the first 0-20nsec. The protein backbone and ligand fluctuations stayed within the range of 0.6 Å and 1.3 Å over the last 80nsec. The amino acid residue VAL135 formed a 95% direct hydrogen bonding interaction with the amide carbonyl, the amino acid residue VAL135 formed 42% direct hydrogen bonding interaction with imidazole in the ring structure, the amino acid residue TYR134 had 33% direct hydrogen bonding interaction, and ARG141 had a 72% PI-cation interaction with 1H-imidazole. The amide carbonyl was exposed to an H _2_O molecule through which it interacted with residue ILE62 (49%), as shown in Figure 2; however, it was reported in **13** triads (previous literature) that the amide carbonyl was exposed to water-mediated contacts with residues such as ILE62 and GLN185 for the IMID2 core scaffold. The 16-triad isopropoxyl group confirmed the probable water molecule interaction with the residue ASP200. The **17** triads showed an indirect interaction between the ortho substituent and residue ASN186. In the 18 triad, the di-F phenyl group was orientated towards LYS85, thus promoting an electrostatic interaction. However, VAL135 interaction formed direct hydrogen bonding interactions with amide carbonyl in all 13, 16, 17, and 18 trial compounds of previous IMID2 scaffolds. ^13^

Interaction diagram of 6Y9R with IMID2 compound code PV-002589535286 was observed during RMSD, Ligand interaction with amino acid residues of Protein Contact during Molecular dynamics simulation, protein–ligand contacts (cont.) (On the x-axis trajectory frame, the number is present, and amino acid residues are seen on the y-axis. The amino acid residues were in greater contact with ligands in the trajectory frame that appeared as a dark color shade). The VAL135 interaction fraction approached 1.4, whereas the ASP133 interaction fraction was missing in comparison to the core IMID2 scaffold from the previous literature. Protein root mean square fluctuation (RMSF) is mostly helpful for predicting changes that occur locally along the enzyme chain. These peaks provide information on how much the protein fluctuated during the molecular dynamic simulation study.

**Figure 3.**
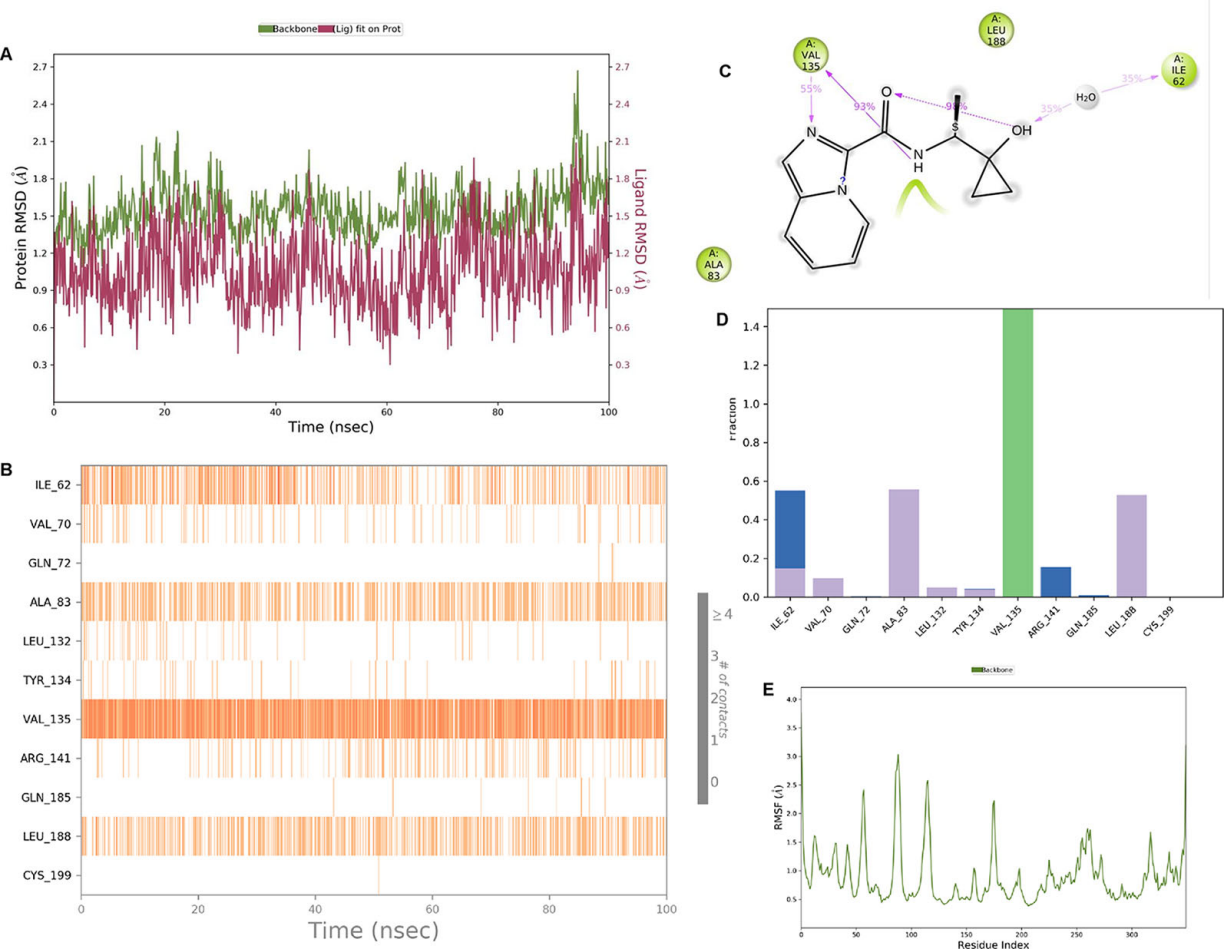
Pictorial representation of 6Y9R interaction with the IMID2 compound code Z3603284828 monitored during the course of MD Simulation trajectory (A) Protein-Ligands RMSD; (B) Protein-Ligand Contacts; (C) Ligand-Protein Contacts; (D) Protein-Ligand Contacts described as histogram; (E) Protein RMSF.

**IMID2 compound code Z3603284828:** During the MD simulations, the ligand-protein complex exhibited hydrogen-bonded interactions. The protein backbone and ligand structure exhibited higher RMSD fluctuations during the first 20 ns. The protein backbone and ligand fluctuations stayed within the range of 0.3 Å and 0.6 Å over the last 70 nsec shown in Figure 3. The amino acid residue VAL135 formed 93% direct hydrogen bonding interaction with the amide carbonyl, the amino acid residue VAL135 formed 55% direct hydrogen bonding interaction with imidazole, and the amino acid residue ILE62 formed 35% water-mediated direct hydrogen bonding interaction with 1-hydroxycyclopropyl.

Interaction diagram of 6Y9R with IMID2 compound code Z3603284828 observed during RMSD, Ligand interaction with amino acid residues of Protein Contact during Molecular dynamics simulation, protein–ligand contacts (cont.) (On the x-axis, a trajectory frame with a number is present, and amino acid residues are seen on the y-axis. The amino acid residues that were in greater contact with ligands in the trajectory frame appeared as a dark color shade). The VAL135 interaction fraction approached 1.4, whereas the ASP133 interaction fraction was missing in comparison to the core IMID2 scaffold from previous literature, and protein RMSF is mostly helpful for predicting the changes that occur locally along the enzyme chain. These peaks provide information on how much the protein fluctuated during the molecular dynamic simulation study.

**Figure 4.**
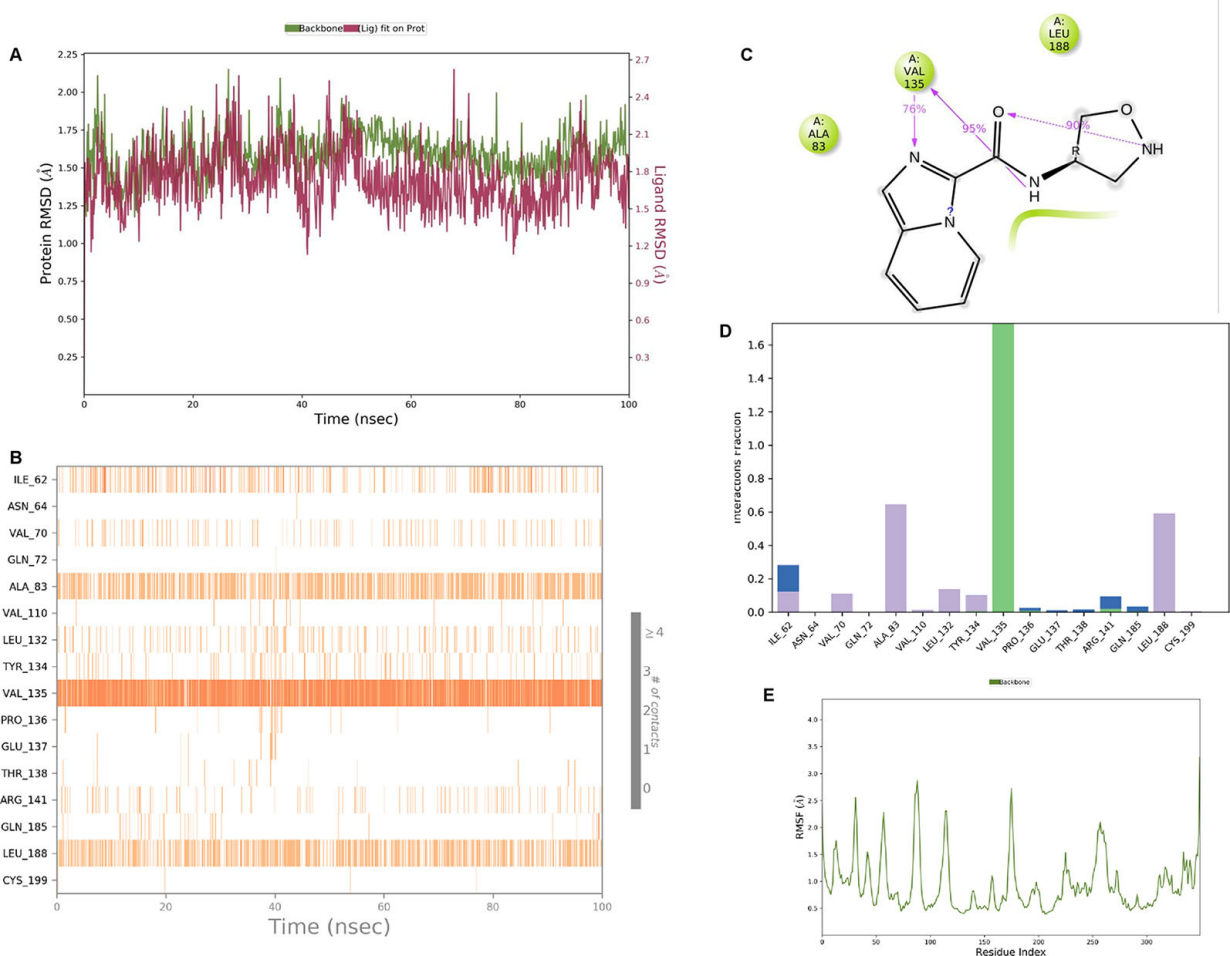
Pictorial representation of 6Y9R interaction with the IMID2 compound code Z3136169169 monitored during the course of Molecular Dynamic Simulation trajectory (A) Protein-Ligand RMSD; (B) Protein-Ligands Contacts; (C) Ligand-Protein Contacts; (D) Protein-Ligand Contacts described as histogram; (E) Protein RMSF.

**IMID2 compound code Z3136169169:** During the MD simulations, the ligand-protein complex exhibited hydrogen-bonded interactions. The protein backbone and ligand structure exhibited higher RMSD fluctuations during the first 20 ns. The protein backbone and ligand fluctuations stayed within the range of 0.8 Å and 0.3 Å over the last 70 nsec shown in Figure 4. The amide carbonyl is exposed to hydrogen bonding and interacts with residue VAL135, and the amino acid residue VAL135 forms 76% direct hydrogen bonding interactions with imidazole.

Interaction diagram of 6Y9R with IMID2 compound code Z3136169169 observed during RMSD, Ligand interaction with amino acid residues of Protein Contact during Molecular dynamics simulation, protein–ligand contacts (cont.) (On the x-axis trajectory frame, the number is present, and amino acid residues are seen on the y-axis. The amino acid residues that were in greater contact with ligands in the trajectory frame appeared as a dark color shade). The VAL135 interaction fraction approached 1.6, whereas the ASP133 interaction fraction was missing in comparison to the core IMID2 scaffold from previous literature and protein RMSF is mostly helpful for predicting the changes that occur locally along with the enzyme chain. These peaks provide information on how much the protein fluctuated during the molecular dynamic simulation study.

**Figure 5.**
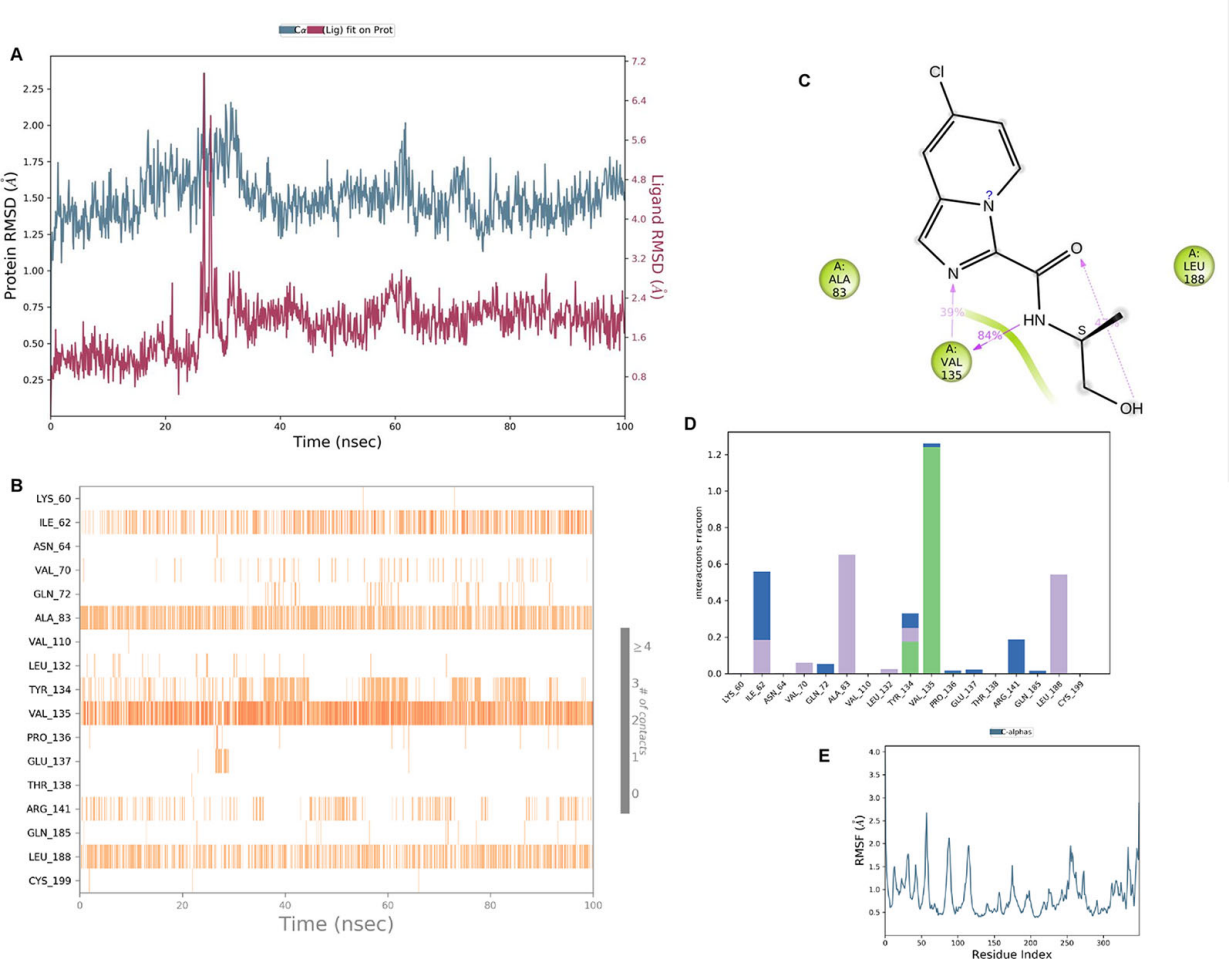
Pictorial representation of 6Y9R interaction with the IMID2 compound code Z2497631540 monitored during the course of MD Simulation trajectory (A) Protein-Ligand RMSD; (B) Protein-Ligand Contacts; (C) Ligand-Protein contacts; (D) Protein-Ligand Contacts described as histogram; (E) Protein RMSF.

**IMID2 compound code Z2497631540:** During the MD simulations, the ligand-protein complex exhibited hydrogen-bonded interactions. The protein Cα and ligand structures exhibited higher RMSD fluctuations over the first 20 ns. The protein backbone and ligand fluctuations stayed within the range of 0.4 Å and 3.7 Å over the last 70 nsec shown in Figure 5. The amino acid residue VAL135 formed 84% direct hydrogen bonding interactions with the amide carbonyl, and the amino acid residue VAL135 formed 39% direct hydrogen bonding interactions with imidazole in the ring structure.

Interaction diagram of 6Y9R with IMID2 compound code Z2497631540 observed during RMSD, Ligand interaction with amino acid residues of Protein Contact during Molecular dynamics simulation, protein–ligand contacts (cont.) (On the x-axis, a trajectory frame with a number is present, and amino acid residues are seen on the y-axis. The amino acid residues that were in greater contact with ligands in the trajectory frame appeared as a dark color shade). The VAL135 interaction fraction approached 1.2, whereas the ASP133 interaction fraction was missing in comparison to the core IMID2 scaffold from previous literature, and protein RMSF is mostly helpful for predicting the changes that occur locally along the enzyme chain. These peaks provide information on how much the protein fluctuated during the molecular dynamic simulation study.

**Figure 6.**
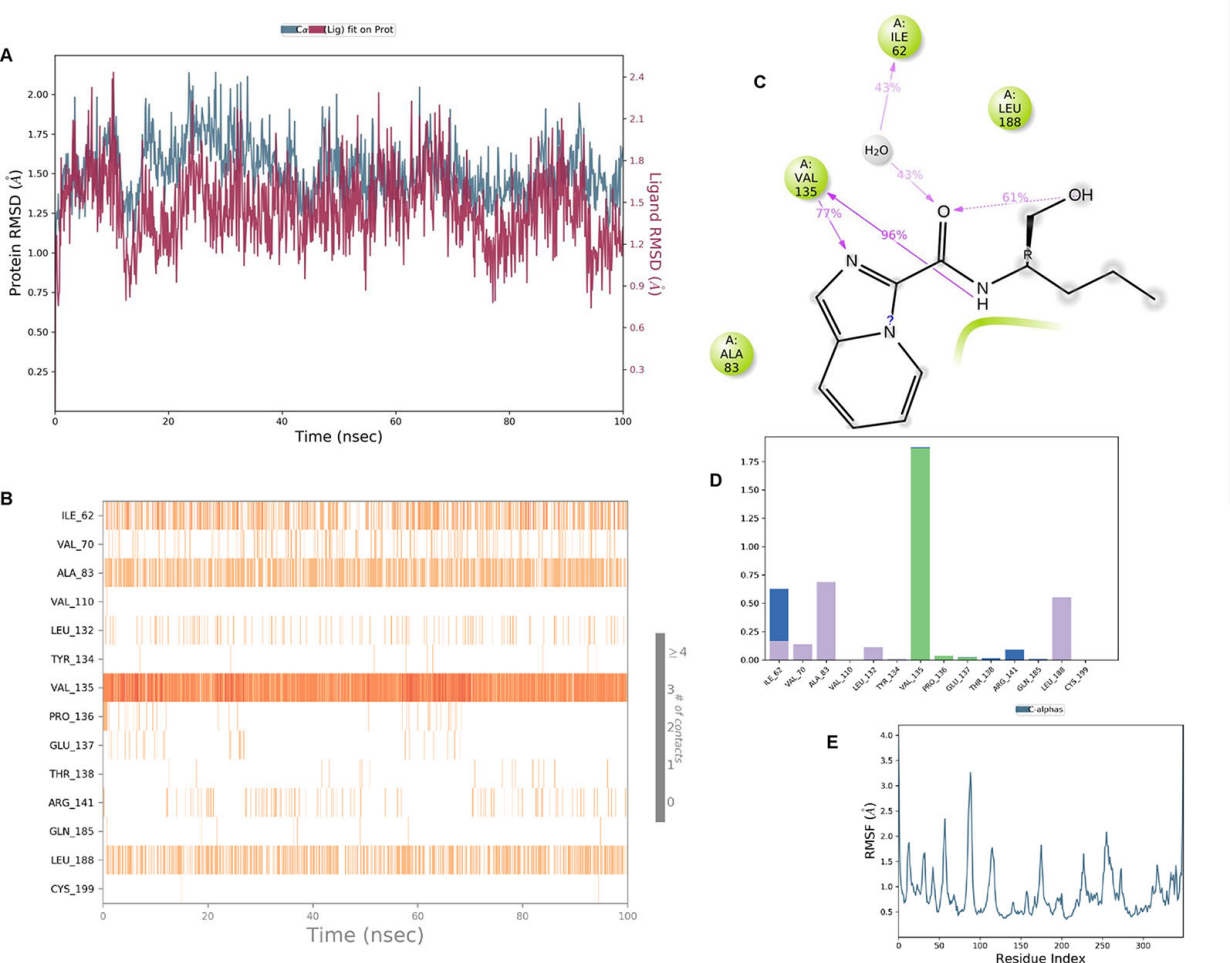
Pictorial representation of 6Y9R interaction with the IMID2 compound code Z4468779454 monitored during the course ofMolecular Dynamic Simulation trajectory (A) Protein-Ligand RMSD; (B) Protein-Ligand contacts; (C) Ligand-Protein contacts; (D) Protein-Ligand contacts described as histogram; (E) Protein RMSF.

**IMID2 compound code Z4468779454:** During the MD simulations, the ligand-protein complex exhibited hydrogen-bonded interactions. The protein Cα and ligand structures exhibited higher RMSD fluctuations over the first 20 ns. The protein backbone and ligand fluctuations stayed within the range of 0.5 Å and 0.4 Å over the last 70 nsec shown in Figure 6. The amino acid residue VAL135 formed a 96% direct hydrogen bonding interaction with the amide carbonyl, the amino acid residue VAL135 formed a 77% direct hydrogen bonding interaction with imidazole, and the amide carbonyl was exposed to the H _2_O molecule through which it interacted with residue ILE62, with 43% and 61% interaction with 1-hydroxypentan-2-yl.

Interaction diagram of 6Y9R with IMID2 compound code Z4468779454 observed during RMSD, Ligand interaction with amino acid residues of Protein Contact during Molecular dynamics simulation, protein–ligand contacts (cont.) (On the x-axis trajectory frame, the number is present, and amino acid residues are seen on the y-axis. The amino acid residues were in greater contact with ligands in the trajectory frame that appeared as a dark color shade). The VAL135 interaction fraction approached 1.75, whereas the ASP133 interaction fraction was missing in comparison to the core IMID2 scaffold from previous literature, and protein RMSF is mostly helpful for predicting the changes that occur locally along the enzyme chain. These peaks provide information on how much the protein fluctuated during the molecular dynamic simulation study.

**Figure 7.**
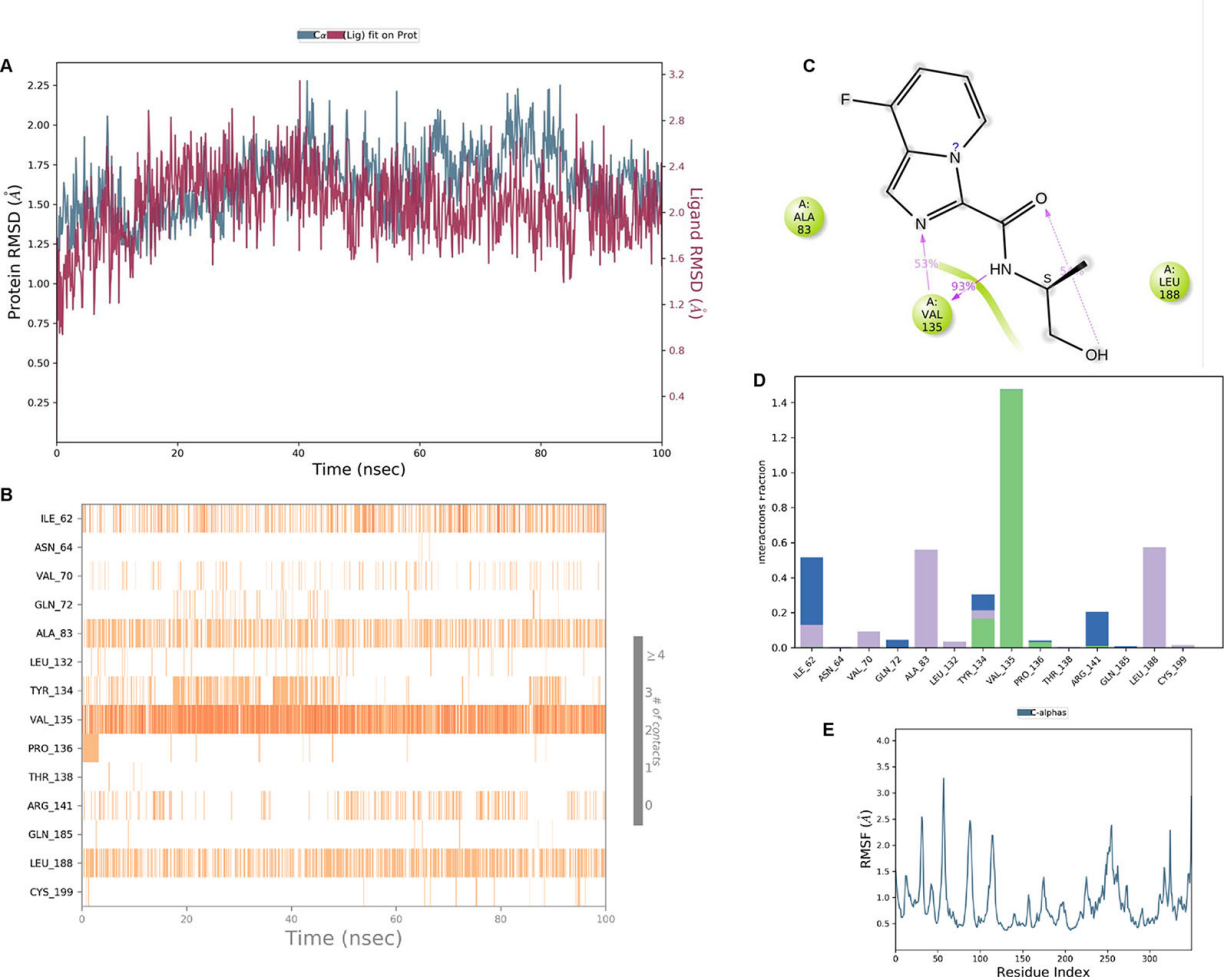
Pictorial representation of 6Y9R interaction with the IMID2 compound code Z2708235883 monitored during the course of MD Simulation trajectory (A) Protein-Ligand RMSD; (B) Protein-Ligand Contacts; (C) Ligand-Protein Contacts; (D) Protein-Ligand Contacts described as histogram; (E) Protein RMSF.

**IMID2 compound code Z2708235883:** During the MD simulations, the ligand-protein complex exhibited hydrogen-bonded interactions. The protein backbone and ligand structure exhibited higher RMSD fluctuations during the first 20 ns. The protein Cα and ligand fluctuations stayed within the range of 0.5 Å and 0.2 Å over the last 70 nsec. The amino acid residue VAL135 formed 93% direct hydrogen bonding interactions with the amide carbonyl, and the amino acid residue VAL135 formed 53% direct hydrogen bonding interactions with imidazole in the ring structure, as shown in Figure 7.

Interaction diagram of 6Y9R with IMID2 compound code Z2708235883 observed during RMSD, Ligand interaction with amino acid residues of Protein Contact during Molecular dynamics simulation, protein–ligand contacts (cont.) (On the x-axis trajectory frame, the number is present, and amino acid residues are seen on the y-axis. The amino acid residues that have been in more contact with ligands in the trajectory frame appear to have a dark color shade). The VAL135 interaction fraction approached 1.4, whereas the ASP133 interaction fraction was missing in comparison to the core IMID2 scaffold from previous literature, and protein RMSF is mostly helpful for predicting the changes that occur locally along the enzyme chain. These peaks provide information on how much the protein fluctuated during the molecular dynamic simulation study.

**Figure 8.**
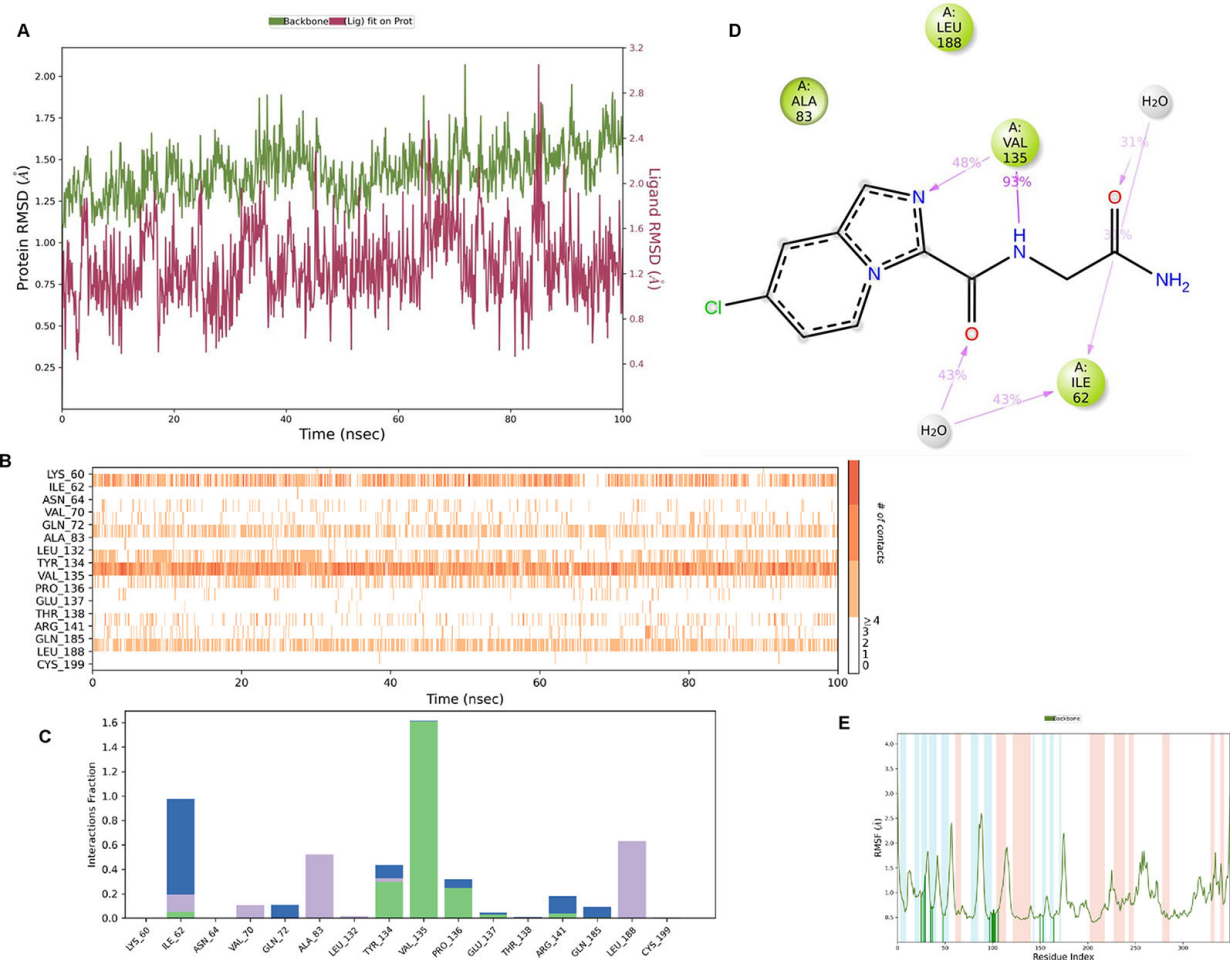
Pictorial representation of 6Y9R interaction with the IMID2 compound code Z3336252116 monitored during the course of MD Simulation trajectory (A) Protein-Ligand RMSD; (B) Protein-Ligand Contacts; (C) Protein-Ligand Contacts described as histogram; (D) Ligand-Protein Contacts; (E) Protein RMSF.

**IMID2_compound code Z3336252116**: During MD simulations, the ligand-protein complex showed hydrogen-bonded interactions. The protein backbone and ligand structure exhibited higher RMSD fluctuations over the first 0-20nsec. The protein backbone and ligand fluctuations stayed within the range of 0.6 Å and 2.2 Å over the last 80nsec. The amino acid residue VAL135 formed 93% direct hydrogen bonding interactions with the amide carbonyl, and the amino acid residue VAL135 formed 48% direct hydrogen bonding interactions with imidazole in the ring structure, which was exposed to the H _2_O molecule through which it interacted with residue ILE62 (43%), as shown in Figure 8.

Interaction diagram of 6Y9R with IMID2_compound code Z3336252116 observed during RMSD, Ligand interaction with amino acid residues of Protein Contact during Molecular dynamics simulation, protein–ligand contacts (cont.) (On the x-axis trajectory frame, the number is present, and amino acid residues are seen on the y-axis. The amino acid residues that have been in more contact with ligands in the trajectory frame appear to have a dark color shade). The VAL135 interaction fraction approached 1.5, whereas the ASP133 interaction fraction was missing in comparison to the core IMID2 scaffold from the previous literature. Protein RMSF is mostly helpful for predicting changes that occur locally along the enzyme chain. These peaks provide information on how much the protein fluctuated during the molecular dynamic simulation study.

**Figure 9.**
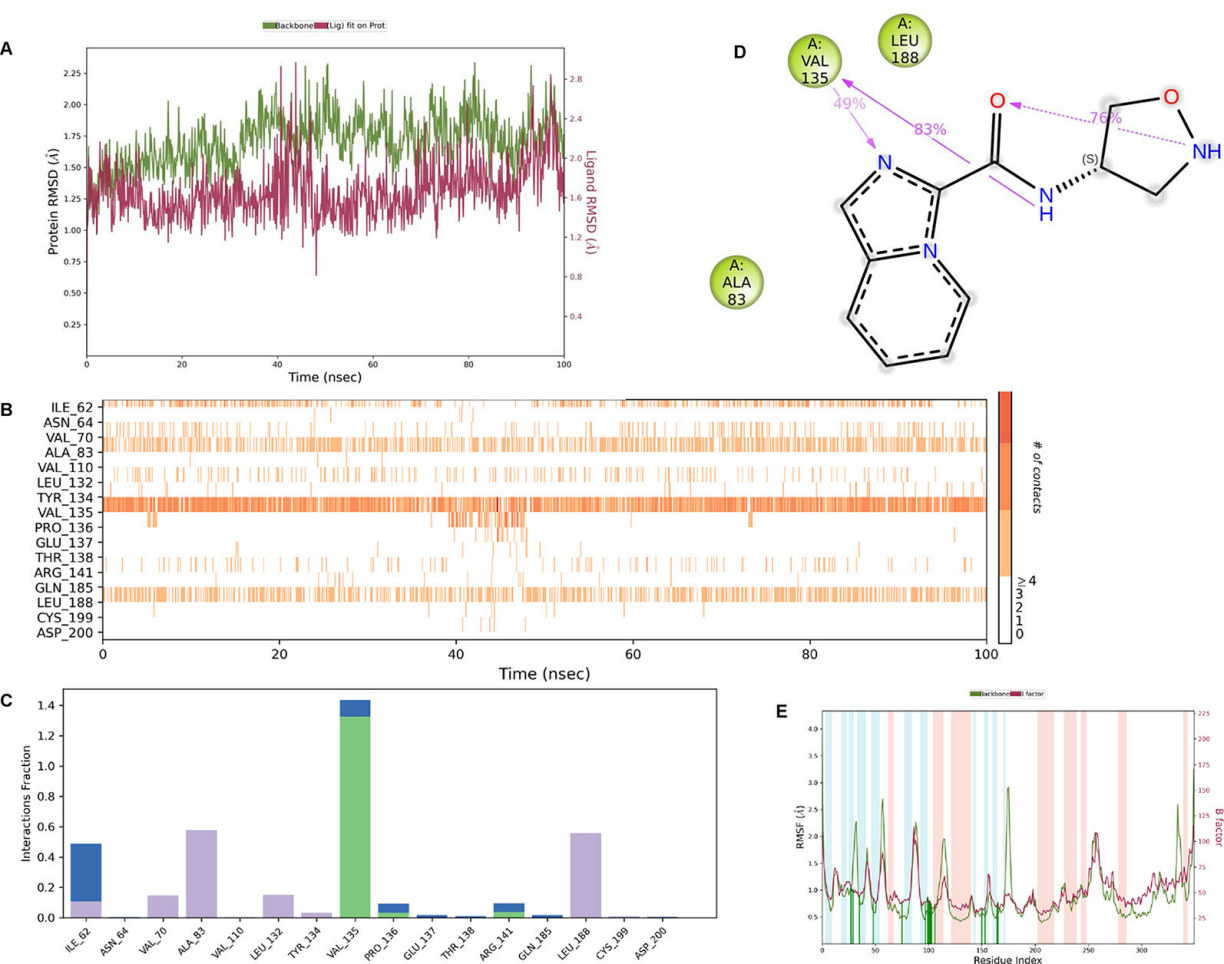
Pictorial representation of 6Y9R interactions with the IMID2 compound code Z3136198649 monitored during the course of Molecular Dynamic Simulation trajectory (A) Protein-Ligands RMSD; (B) Protein-Ligand Contacts; (C) Protein-Ligand Contacts described as histogram; (D) Ligand-Protein Contacts; (E) Protein RMSF.

**IMID2 compound code Z3136198649**: During MD simulations, the ligand-protein complex showed hydrogen-bonded interactions. The protein backbone and ligand structure exhibited higher RMSD fluctuations over the first 0-20nsec. The protein backbone and ligand fluctuations stayed within the range of 0.7 Å and 0.5 Å over the last 80nsec. The amino acid residue VAL135 formed 83% direct hydrogen bonding interaction with amide carbonyl, and the amino acid residue VAL135 formed 49% direct hydrogen bonding interaction with imidazole in the ring structure given in Figure 9.

Interaction diagram of 6Y9R with IMID2 compound code Z3136198649 observed during RMSD, Ligand interaction with amino acid residues of Protein Contact during Molecular dynamics simulation, protein–ligand contacts (cont.) (On the x-axis trajectory frame, the number is present, and amino acid residues are seen on the y-axis. The amino acid residues that have been in more contact with ligands in the trajectory frame appear to have a dark color shade). The VAL135 interaction fraction approached 1.4, whereas the ASP133 interaction fraction was missing in comparison to the core IMID2 scaffold from the previous literature. Protein RMSF is mostly helpful for predicting changes that occur locally along the enzyme chain. These peaks provide information on how much the protein fluctuated during the molecular dynamic simulation study.

**Figure 10.**
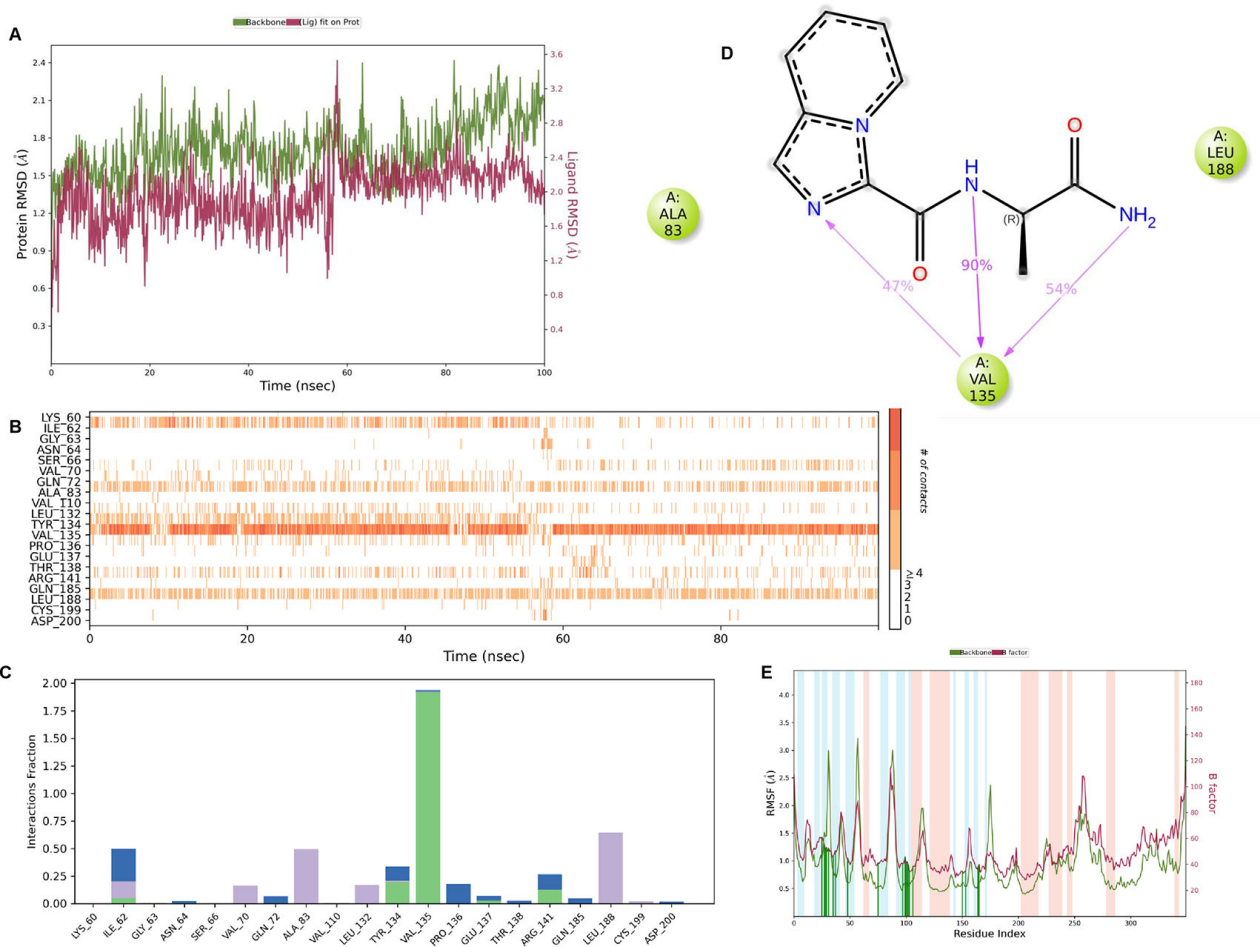
Pictorial representation of 6Y9R interaction with the IMID2 compound code PV-005996498401 monitored during the course of Molecular Dynamic Simulation trajectory (A) Protein-Ligand RMSD; (B) Protein-Ligand contacts; (C) Protein-Ligand contacts described as histogram; (D) Ligand-Protein contacts; (E) Protein RMSF.

**IMID2 compound code PV-005996498401**: During MD simulations, the ligand-protein complex showed hydrogen-bonded interactions. The protein backbone and ligand structure exhibited higher RMSD fluctuations over the first 0-20nsec. The protein backbone and ligand fluctuations stayed within the range of 1.0 Å and 0.6 Å over the last 80nsec. The amino acid residue VAL135 formed 90% direct hydrogen bonding interactions with the amide carbonyl, and the amino acid residue VAL135 formed 47% direct hydrogen bonding interactions with imidazole in the ring structure, as shown in Figure 10.

Interaction diagram of 6Y9R withIMID2 compound code PV-005996498401 observed during RMSD, Ligand interaction with amino acid residues of Protein Contact during Molecular dynamics simulation, protein–ligand contacts (cont.) (On the x-axis trajectory frame, the number is present, and amino acid residues are seen on the y-axis. The amino acid residues that have been in more contact with ligands in the trajectory frame appear to have a dark color shade). The VAL135 interaction fraction approached 1.85, whereas the ASP133 interaction fraction was missing in comparison to the core IMID2 scaffold from the previous literature. Protein RMSF is mostly helpful for predicting changes that occur locally along the enzyme chain. These peaks provide information on how much the protein fluctuated during the molecular dynamic simulation study.

## Conclusion

In this study, we used focused library generation to target the receptor grid region (ATP-competitive site) of 6Y9R. After docking, the compounds were separated and selected based on the VAL135 residue interaction. Further prediction was performed using Qikprop and Prime MM-GBSA assays and Molecular dynamic simulation studies. Further experimental studies are required to confirm these findings.

### Ethical considerations

Ethics and written consent were not applicable.

## Data Availability

Figshare: Molecular docking studies and molecular dynamic simulation to identify GSK-3β inhibitors for Alzheimer’s disease, https://doi.org/10.6084/m9.figshare.24592716.v1. ^24^ The underlying data for this project are:
➢
Enamine_IMID2_Scaffold.csv ➢
Enamine_IMID2_Scaffold_with nitrogen_realdb_molecules 17_35_52.sdf ➢
Ligprep_enamine_IMID2_Scaffold_withnitrogen_realdb_out.csv ➢
Ligprep_enamine_IMID2_SCAFFOLD_WITHNITROGEN_REALDB-out.mae ➢
Prime_mmgbsa_3_out.csv ➢
prime_mmgbsa_3-out.maegz ➢
Prime_mmgbsa_VAL135_INTERACTION_IMID2_ENAMINE_WITHNITROGEN-out.csv ➢
prime_mmgbsa_VAL135_INTERACTION_IMID2_ENAMINE_WITHNITROGEN-out.maegz ➢
QIKPROP_LIGPRE_IMID2_ENAMINE_WITHNITROGEN-out.csv ➢
QIKPROP_LIGPREP_ENAMINE_IMID2_SCAFFOLD_WITHNITROGEN_REALDB-out.mae Data are available under the terms of the Creative Commons Attribution 4.0 International license (CC-BY 4.0). Protein Data Bank: 6Y9R, https://doi.org/10.2210/pdb6Y9R/pdb. ^25^
