# Molecular docking studies and molecular dynamic simulation analysis: To identify novel ATP-competitive inhibition of Glycogen synthase kinase-3β for Alzheimer’s disease

**DOI:** 10.12688/f1000research.145391.3

**Published:** 2025-05-27

**Authors:** Suggala Ramya Shri, Yogendra Nayak, Sreedhara Ranganath Pai

**Affiliations:** 1 Department of Pharmacology, Manipal College of Pharmaceutical Sciences, Manipal Academy of Higher Education, Manipal, Karnataka, 576104, India

**Keywords:** Alzheimer’s disease, GSK-3β, ATP-competitive study, Protein preparation, Ligand Preparation, Qikprop, Glide module, Prime MM-GBSA, Molecular dynamic simulation.

## Abstract

**Background:**

The discovery of an ideal and effective therapy is urgently required for the treatment of Alzheimer’s disease (AD). The main pathological hallmarks of Alzheimer’s disease that appear before the clinical symptoms are neurofibrillary tangles, amyloid plaques, brain inflammation, and neuronal atrophy throughout the cerebral cortex and hippocampus. GSK-3β (Glycogen Synthase Kinase-3β) is regarded as the most important and promising target for therapeutic use because GSK-3β expression levels increase with age and are the most abundant and hyperactive in the brains of patients with AD. GSK-3β activation or upregulation can contribute to neurodegeneration by promoting amyloid beta (Aβ) production and tau hyperphosphorylation. Whereas the underlying mechanism for abnormal production of GSK-3β in AD brains remains unclear.

**Methods:**

Maestro was used, which is Schrodinger, for our computational simulation studies. In the present work, different modules that were used in previous studies with a little modification, the modules such as Protein Preparation with the help of Protein Preparation Wizard, Ligand Preparation with the help of LigPrep, for ADME (Absorption, Distribution, Metabolism and Excretion) prediction Qikprop was used, for docking studies Glide module was used, Binding energy prediction the Prime was used and Molecular dynamic simulation (MDs) studies done using Desmond.

**Results:**

Our focus is mainly on an *in-silico* approach, focusing on library generation; first draw an IMID2 (imidazo [1,5-a]pyridine-3-carboxamide) scaffold structure at Enamine and subjected it to a substructure search to target the receptor grid region (ATP-competitive site) of 6Y9R. They were then subjected to various screening processes. Finally, nine compounds were subjected to MDs studies.

**Conclusions:**

Nine compounds showed good results with the most stable interactions. Among all the MD studies, the compound (Z3336252116) has shown good interaction and a good docking score. Further experiments and studies are required to confirm these results.

## Introduction

One of the greatest threats to public health is neurodegenerative diseases because there is no exact therapy. Therefore, the discovery of an ideal and effective therapy is urgently needed for the treatment of AD. ^1^ Only five to seven cases are due to genetic mutations, and the remaining cases are due to environmental factors and sporadic mutations. The main pathological hallmarks of AD that appear before the clinical symptoms are neurofibrillary tangles, amyloid plaques, brain inflammation, and neuronal atrophy throughout the cerebral cortex and hippocampus. ^2^ Memory loss can be elicited in Alzheimer’s patients, such as episodic short-term memory impairment followed by a lack of motivation, disorganization, and impairment in solving problems, judgment, and executive functioning. In early-stage impairments, such as visuospatial skills, neuropsychiatric symptoms are most common in mild and late-stage AD. ^3^ The heritability of AD is estimated to be between 60% and 80%, and these components allow for the identification and determination of pathophysiological processes, diagnostic markers, biological targets, and new treatment targets through genomics translational studies. ^4^ The aducanumab, a medication based on the Aβ theory, in 2021 FDA approved for the treatment of AD. ^5^ Lecanemab’s effectiveness and safety in treating early-stage AD require longer study trials. ^6^ AD is the leading cause of dementia among the elderly, with an estimated 44 million individuals affected globally, a number projected to double by 2050. In the U.S., over 5.5 million are currently diagnosed. The pathophysiology of AD includes several key features: the presence of amyloid plaques, which disrupt neuronal communication and incite inflammatory responses; neurofibrillary tangles formed by hyperphosphorylated tau protein, leading to neuronal dysfunction and cell death; and neuroinflammation, where activated glial cells further damage neurons and exacerbate the disease’s progression. Additionally, AD is characterized by neurotransmitter dysregulation, notably a marked decline in acetylcholine levels affecting cognitive abilities, and imbalances in glutamate neurotransmission, which leads to synaptic dysfunction and potential excitotoxicity. ^7^^,^^8^

GSK-3 (Glycogen synthase kinase-3) is a member of the protein kinase family and is widely expressed in tissues. GSK-3 is a Serine/Threonine kinase that transfers a phosphate group to either the serine or threonine residue of its substrate target. ^9^

GSK-3 is a significant player in regulating structure and metabolic processes in developing neurons as well as adult neurons, but the nervous system can contribute to the disease pathogenesis because overexpression of GSK-3 can contribute to the progressive neurodegenerative conditions like Alzheimer’s. ^10^^–^^12^

Overactivation or inhibition of GSK-3 activity in the brain influences sociability skills, mood, emotion, and schizophrenia behavior. ^13^^–^^17^ Whereas the reduced activity of GSK-3 can decrease or reverse the severity of a large number of diseases.

GSK-3β activation or upregulation can contribute to neurodegeneration by promoting Aβ production and tau hyperphosphorylation whereas the Aβ induces neuronal damage and tau hyperphosphorylation leading to impair the formation of neurofibrillary tangles (NFTs) and their synaptic dysfunction in AD. In general, GSK-3β is highly expressed in the Central Nervous System (CNS) and regulates the hippocampal neurogenesis and biological response of primary immune cells of CNS, microglia, synaptic plasticity, learning, and memory. Activation of GSK-3β kinase has been associated with the inhibition of hippocampal LTP. This hippocampal mechanism is needed for memory formation. ^18^

GSK-3β is highly expressed in the CNS and its activity is increased in the brain and plasma of AD patients. ^19^^–^^24^ Abundant evidence suggests that the inhibition of GSK3β improves both synaptic and cognitive function in AD mouse model studies ^25^^–^^29^ and useful therapy for a large of neurological diseases or disorders. The underlying mechanism for abnormal production of GSK-3β in AD remains unclear. ^30^

Long-term depression (LTD) and long-term potentiation (LTP) are important for regulating the synaptic connections between neurons. ^31^ The synaptic dysfunction is an early sign of AD. ^32^ The LTP mainly involves excitotoxicity, plasticity, synaptic formation, learning and memory. The LTP is generally defined as the long-lasting increase in synaptic strength, whereas LTD refers to an opposing process. ^33^ GSK-3β is expressed in brain regions such as the hippocampus which is involved in learning, and memory and plays an important role in regulating the balance (LTP and LTD). The induction of LTD is associated with a decrease in the phosphorylation of GSK-3β at SER9, whereas the induction of LTP can prevent the LTD occurrence. Finally, inhibiting the active form of GSK-3β is useful for the induction of LTP. ^34^ Presenilin 1(PS1), a product of the PSEN1 gene, is an important causative factor for familial Alzheimer’s Disease (FAD), and also plays a key role in cleaving APP. ^35^ GSK-3β-mediated PS1 phosphorylation affects its interaction with N-cadherin and disrupts their binding interaction, finally leading to neuronal activity deficits and synaptic functioning deficits. ^36^ The interplay among GSK-3β, PS1, and its impact on synaptic functioning deficits and Aβ metabolism can contribute to the pathogenesis of AD.

GSK-3β is regarded as the most important and promising therapeutic target. The etiology and pathogenesis of AD are not completely understood. However, available treatments have failed to show novel approaches and effectiveness, and the efficacy of drugs varies from one person to another. ^37^ GSK-3β expression levels increase with age and are most abundant and hyperactive in the brains of patients with AD. Hence, dysregulation of GSK-3β automatically affects amyloid beta plaques, which have been previously shown in in vitro and in vivo AD models. ^7^^,^^38^ GSK-3 plays a very important key role in the metabolic process and regulating structural processes in adult neurons as well as in developing neurons. ^39^ In the present study, molecular modeling approach was used. For better BBB (Blood Brain Barrier) permeation, the structure was finalized based on a wet lab. So, based on the literature search, a core imidazole scaffold was selected for this study, and from that core imidazole scaffold, the substructures was drawn in the enamine database. Then docking studies were done on the compounds and subjected to ADME, whereas the ADME was predicted with the help of Qikprop. MDs study was conducted on nine compounds.

## Methods

### Software used for this computational study

Maestro was used for our computational simulation studies; the graphical interface was Schrödinger. In the present work, utilised different modules that were used in previous studies.: Protein Preparation Wizard, Ligand Preparation with the help of LigPrep, for ADME prediction, Qikprop was used, for Docking studies the Glide module was used, and binding energy prediction Prime was used and MDs studies was done by Desmond. ^40^^-^^42^ Alternatively freely available software are AutoDock 4, ArgusLab, and Gromacs.

### Selection and preparation of protein

In the present study PDB I.D: 6Y9R ( http://www.rcsb.org/) ^43^ of GSK-3β was selected, which is bound to a co-crystallized ligand with a Resolution of 2.08 Å consisting of only chain A, downloaded from the protein databank site, ^41^^,^^42^ and imported this PDB I. D to the Maestro interface. This is followed by preparing the protein molecule with the help of the protein preparation wizard panel of the Schrödinger suite. Alternatively, the AutoDock 4 which is freely available software can be used. The first step for protein preparation is preprocessing in the workspace the protein structure is selected then Assigned bond orders, using the CCD database, explicit hydrogen to the structure, zero-order bonds to metals, creating disulfide bonds, and optimizing missing side chain atoms by running a Prime job, fill in missing loops by running the Prime job was selected and delete waters that are further than specified distance beyond 5.00 Å from any of the het groups including ions. This is recommended for Glide and virtual screening, but MD applications should keep these water as they will help equilibrate the solvent box faster and generate het states using Epik pH: 7.5±0.0, and clicked for preprocessing. The second step is to review and modify here when a new chain, water, or ht is selected to zoom to fit the selection to the workspace, select waters and hets within 5.0 Å of selected chains, and keep the remaining residues and chains as the default setting and generate a state pH of 7.5±0.0. The third step is to refine the selected sample water orientations in addition to other groups, and the protonation states of residues and ligands were set by using PROPKA pH: 7.5 then it simulated the exact experimental conditions and clicked to automatically optimize hydroxyl, ASN, GLN, HIS states using ProtAssign, removing water that is further than beyond hets 3.0 Å, including ions. This is recommended for Glide and virtual screening, but MD applications should keep this water as it will help equilibrate the solvent box faster. OPLS3e force field for the restrained minimization of proteins was done. Then after the minimization, the glycerol and acetate ions were removed from the minimized protein.

### Ligand selection and preparation

IMID 2 scaffolds were used for this study. This scaffold possesses fewer hydrogen bond donors and improved CNS (Central Nervous System) penetration at micromolar concentrations (μM), based on studies by Buonfiglio et al. ^44^ To generate the focus library, draw an IMID 2 scaffold structure at Enamine and subjected it to a substructure search. Then, enamine realdb_IMID2_Scaffold_molecules (1400 molecules) was downloaded, followed by ligand preparation using LigPrep in the Schrödinger suite software (Schrödinger 2021-3). ^45^ Alternatively, the AutoDock 4 which is freely available software can be used. The 3D coordinates for the compounds were generated using the LigPrep tool, and the Epik module predicted the most probable charge form for the ionization state of compounds at pH: 7.5±0.0, generated tautomers and stereoisomers, determined chiralities from the 3D structure, generated at most 32 per ligand, and finally, the minimization of the drug molecule process was performed using the force field OPLS3e. ^46^^,^^47^

### ADME prediction

After ligand preparation, all ligands were subjected to QikProp in the Schrödinger suite software to predict the ADME profile of the compounds. QikProp predicts the drug-like properties of all selected compounds, such as molecular weight, Hydrogen Bond Donors, Hydrogen Bond Acceptor, QPlogS (predicted aqueous solubility), QPlogPo/w (predicted octanol/water partition coefficient), QPlogBB (predicted brain/blood partition coefficient), QPlogPw (predicted water/gas partition coefficient), QPlogPC16 (predicted hexadecane/gas partition coefficient), QPlogPoct (predicted octanol/gas partition coefficient), PSA (Van der Waals surface area of polar nitrogen and oxygen atoms), QPPCaco (predicted apparent Caco-2 cell permeability), and QPPMDCK (predicted apparent MDCK cell permeability). ^48^

### Molecular docking studies

Here, Schrödinger suite Glide module was used for better prediction of the protein-ligand involved in assessing the fitting of all conformations of compounds at the binding site followed by ranking and modes Schrödinger Release 2021-3. The alternative freely accessible software is AutoDock and Gromacs. The molecular docking study process was used for assessment before molecular docking studies, and the default settings were used for the Receptor Grid generation module. The binding site was recognized using the Receptor Grid generation module, and this binding site was specified as a box of 10 × 10 × 10 Å ^3^ centered on the centroid of the co-crystallized ligand of GSK-3β, which was taken into consideration for grid generation. ^49^ Then subject all prepared compounds to the SP (standard precision) scoring function mode. ^50^ Glide SP docking ligand sampling is in flexible mode and selected sample nitrogen inversions, sample ring conformations, and amides only penalize non-planar conformations, adding Epik state penalties to the docking score. In the output file, the pose viewer file that includes the receptor, written out at most one pose per ligand, performed post-docking minimization, the number of poses per ligand to include was five, and the RMSD was computed to input ligand geometries and run the job. Finally, the results were analyzed, separated, and selected based on the VAL135 residue interaction and physicochemical properties reported in the literature. These compounds were then subjected to the prime-MMGBSA module.

### MM-GBSA binding energy

MM-GBSA (molecular mechanics-generalized born energies & surface area/accessibility continuum solvation method) was used to calculate the binding free energy and binding analysis of 523 SP docked protein-ligand complexes. MM-GBSA exhibits many energy properties for proteins, ligands, and complex structures. Prime MM-GBSA analysis is based on the solvent model of VSGB 2.0, and the OPLS3e force field is used to calculate the binding affinity of the respective protein-ligand complex. ^51^

### MDs with Desmond

This model was used to precisely predict the interaction of the ligand with 6Y9R (protein), the stability of their binding under physiological conditions, and to analyze their motion at the molecular and atomic levels. The protein-ligand complex was subjected to a system built here, and different solvent models were selected; an orthorhombic box with dimensions of 10 Å × 10 Å × 10 Å was used to determine the structure geometry to have the minimum box volume, and the checked boundary box is shown in the workspace. The next step is to load the previous system builder file and enter 100ns in simulation time, 1000frames were captured, ensemble class is NPT (Normal Pressure Temperature), 300°K temperature, pressure bar is 1, and relax model system before simulation was used for MDs studies. ^52^ After MDs, the analysis was done with the help of the RMSD (Root Mean Square Deviation), the RMSD of ligands, RMSD of protein. RMSD is mainly used to determine the stability of the protein-ligand complex, RMSF (Root Mean Square Fluctuations), ligand-protein contacts, and ligand interaction diagram. Alternatively, NAMD molecular simulation along with VMD software can be used which is freely available.

## Results and Discussion

When PDB was searched in UniProt, then 89 GSK-3β human PDBs was identified. Out of 89 PDB entries, 8 PDB literature are yet to be published, and some PDBs with position 3-12 amino acids residual chains C and D are removed from my PDB selection list. PDBs that have chain A/B are taken into consideration, and individually remaining PDBs were studied. Finally, selected this PDB: 6Y9R for this study. ^62^

The co-crystal structure of 2 was in complex with PDB I. D 6Y9R, which is a GSK-3β enzyme. While INDZ was located at the hinge region, the piperidine chain was exposed to the solvent towards ARG141, Pyridine formed a hydrogen bond with LYS85, nearer to the ligand several hydration sites with residues such as THR138, ASN186, GLN185, LEU132, ASP200, and IIE62 were found to be fully explained in a previous study. The INDZ core scaffold was located in the ATP-competitive binding site of GSK-3β between the N-terminal lobe and the C-terminal lobe region of the protein, and INDZ was previously developed as a novel GSK-3β inhibitor using computational tools. The N-2 of the core and VAL135 N-H group, hydrogen at position N-1 of the core with the ASP133 carbonyl group, hydrogen of the carboxamide group, and VAL135 carbonyl group are important interactions. The R1 substituent appended to the core is oriented towards ARG141 in the external solvent-accessible part of the solvent, and the R2 substituent appended to the LYS85 inner cavity proximity. The indazole N-Ha was then replaced with the C-Ha group, and the ring nitrogen atoms were shuffled, resulting in the IMID2 core. ^44^

**Figure 1.**
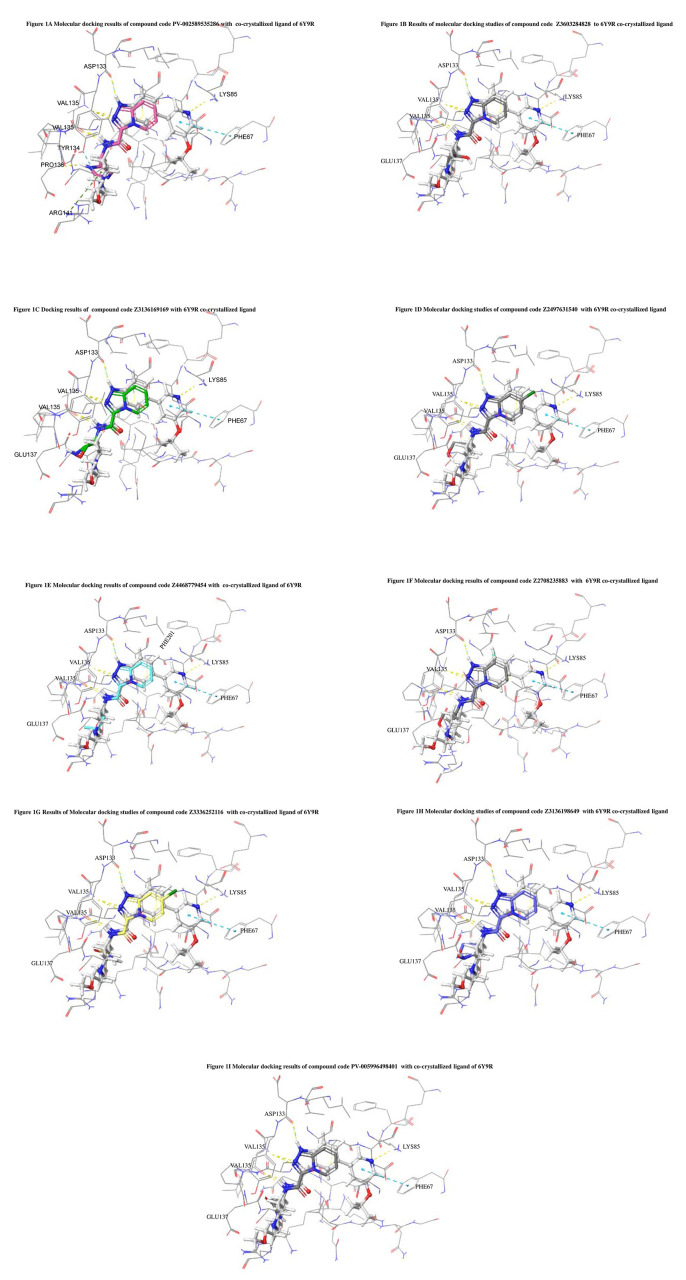
Overlap of co-crystal structure of 6Y9R and IMID2 substructures.

The docked ligands accommodated in the ATP-binding site are similar to the co-crystal structure of the 6Y9R protein. The overlap of the co-crystal structures of the 6Y9R and IMID2 substructures is shown in Figure 1 ( Figures 1A to 1I). In the previous literature it was reported that a highly flexible piperidine group was exposed to GLN72 and a water bridged H-bond which was involving the R1 oxanyl ring was noticed with PRO136.

The docking of ligands to a particular receptor grid generated a region of the protein 6Y9R. In this study, SP docking was used. After docking, the compounds were separated and selected based on the VAL135 residue interaction in the literature. Therefore, based on the VAL135 N-H group {the H group of the VAL135 protein should show direct interaction with the nitrogen group of the ligand}. Through this process, 523 molecules of the IMID2 scaffold was finalized. Compounds with good SP scores are listed in Table 1.

**Table 1.** Intermolecular interactions with amino acids and docking score of the top 9 selected compounds.

Name	Docking score	Structure	Bonded interaction	Non-bonded interaction
Z3336252116	-8.535	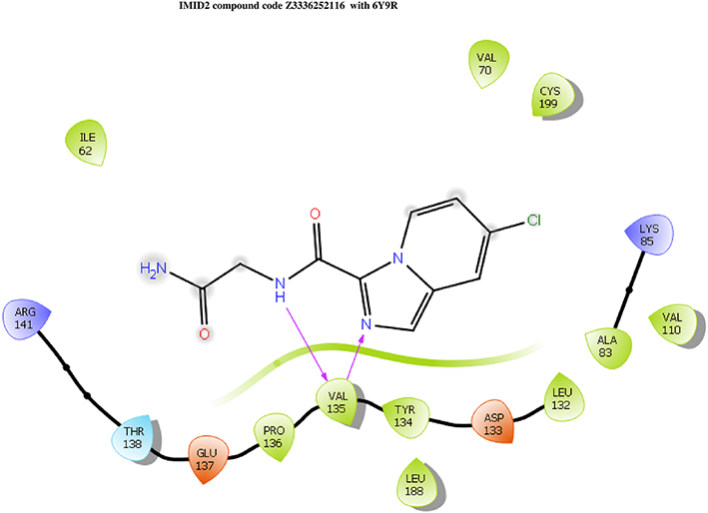	H-bond: VAL135,	ILE62, ARG141, THR138, GLU137, PRO136, TYR134, LEU188, ASP133, LEU132, ALA83, VAL110, LYS85, CYS199, VAL70
Z3136198649	-8.285	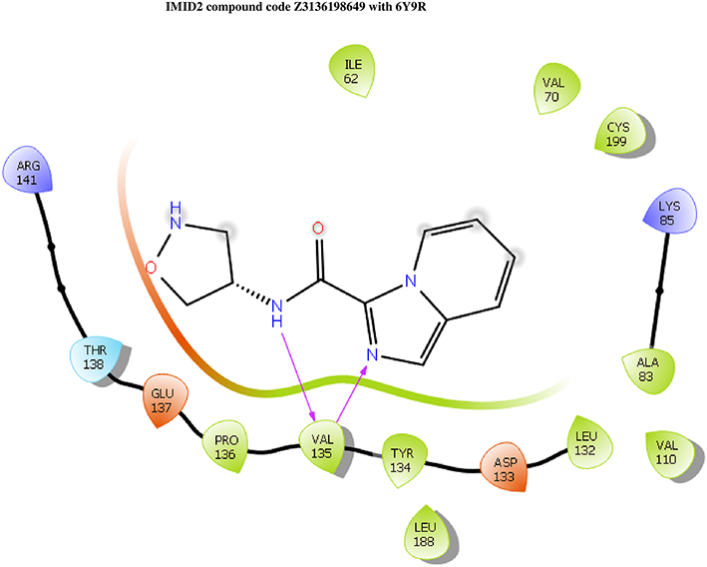	H-bond: VAL135	ILE62, ARG141, THR138, GLU137, PRO136, TYR134, LEU188, ASP133, LEU132, ALA83, VAL110, LYS85, CYS199, VAL70
PV-005996498401	-8.132	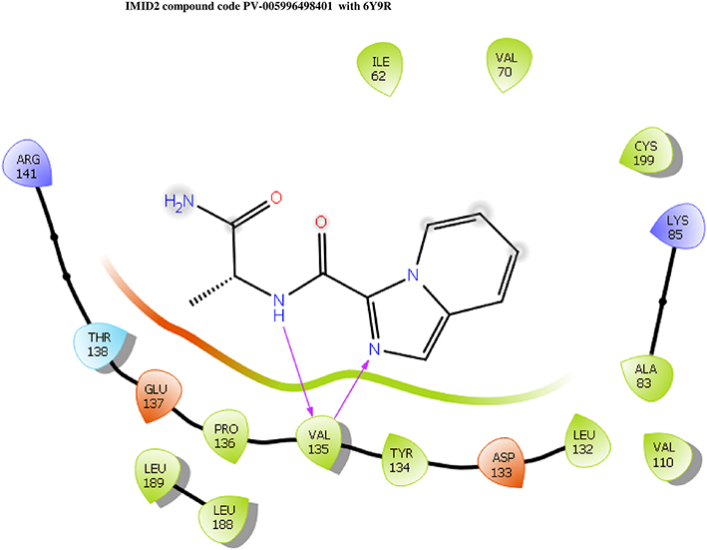	H-bond: VAL135	ARG141, THR138, GLU137, PRO136, LEU189, LEU188, TYR134, ASP133, LEU132, VAL110, ALA83, LYS85, CYS199, VAL70, ILE62
PV-0025895352867	-7.541	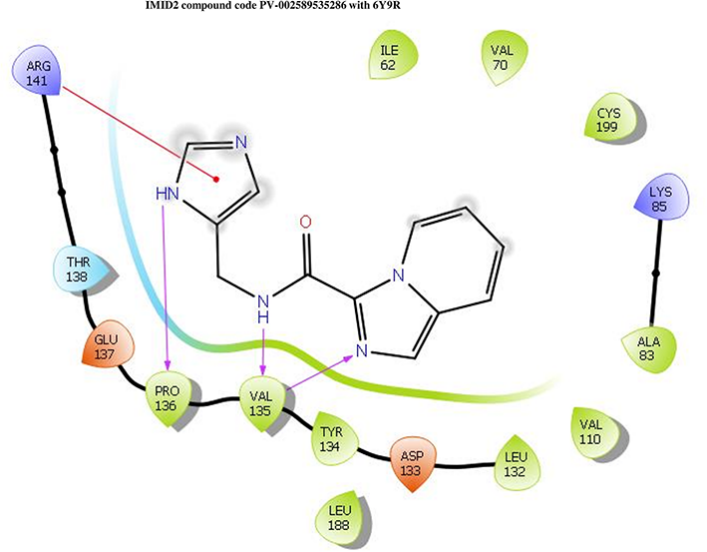	H-bond: VAL135, PRO136 PI-cation: ARG141	VAL110, ALA83, LYS85, CYS199, VAL70, ILE62, LEU132, ASP133, TYR134, LEU188, GLU137, THR138
Z3603284828	-7.765	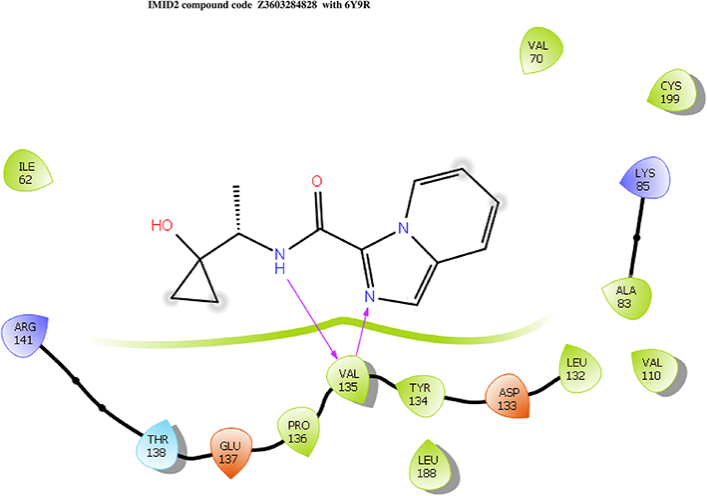	H-bond: VAL135	ILE62, ARG141, THR138, GLU137, PRO136, TYR134, LEU188, ASP133, LEU132, ALA83, VAL110, LYS85, CYS199, VAL70
Z3136169169	-8.268	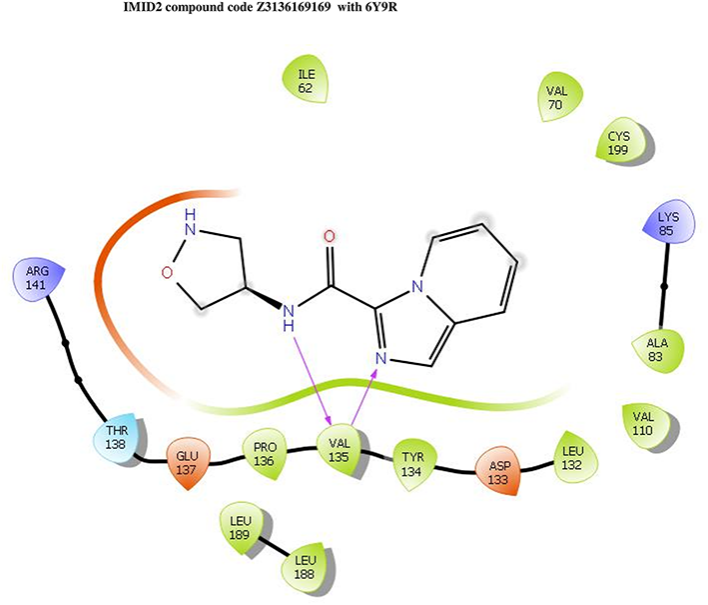	H-bond: VAL135	ARG141, THR138, GLU137, PRO136, LEU189, LEU188, TYR134, ASP133, LEU132, VAL110, ALA83, LYS85, CYS199, VAL70, ILE62
Z2497631540	-7.967	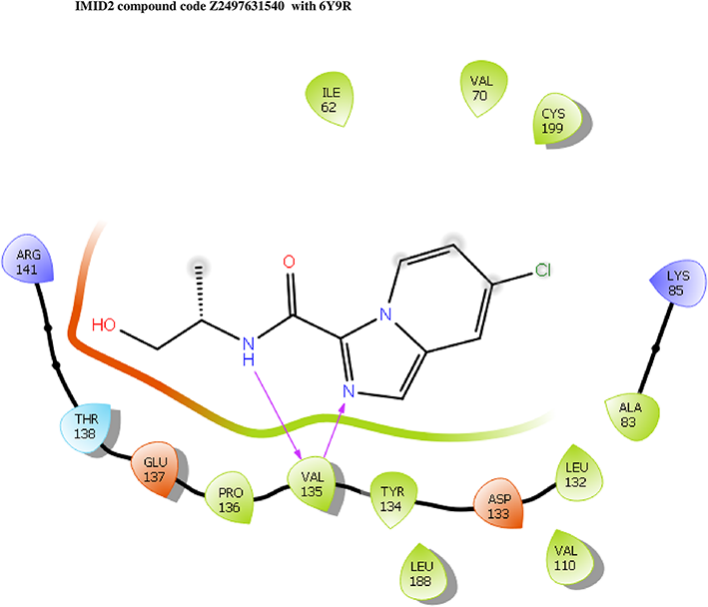	H-bond: VAL135	ARG141, THR138, GLU137, PRO136, TYR134, LEU188, ASP133, LEU132, VAL110, CYS199, ALA83, LYS85, VAL70, ILE62
Z4468779454	-7.306	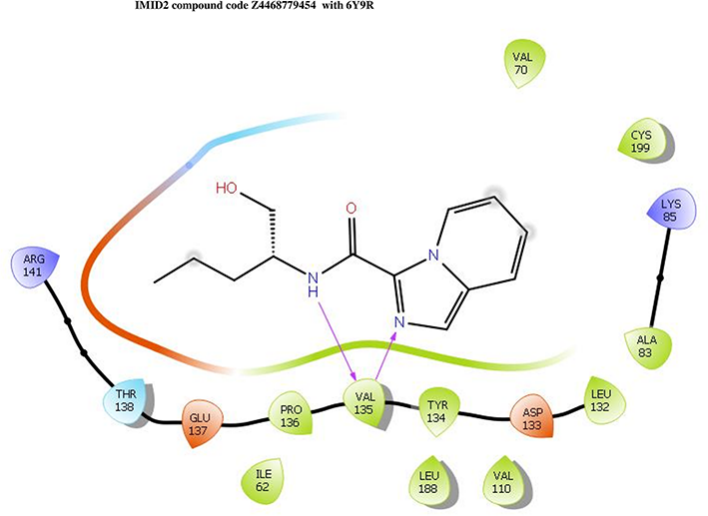	H-bond: VAL135	ARG141, THR138, GLU137, PRO136, TYR134, LEU188, ASP133, LEU132, VAL110, CYS199, ALA83, LYS85, VAL70, ILE62
Z2708235883	-7.961	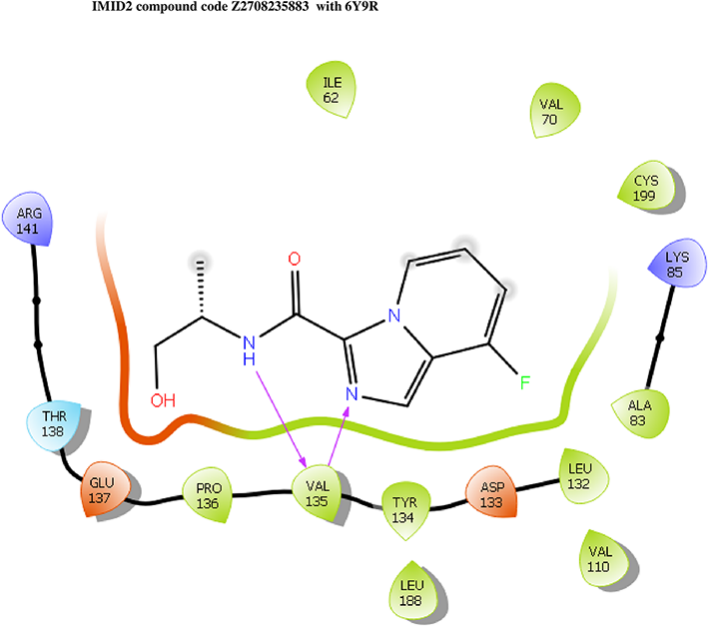	H-bond: VAL135	ARG141, THR138, GLU137, PRO136, TYR134, LEU188, ASP133, LEU132, VAL110, CYS199, ALA83, LYS85, VAL70, ILE62

Among all the compounds, the compound (Z3336252116) has shown good docking score (-8.535 Kcal/mol) and with bonding interaction VAL135 which was similar to previous literature (hydrogen of the N-H group of VAL135).

### ADME prediction

The ADME or drug-likeness parameters are the most important and helpful parameters for screening compounds after SP docking studies during the drug discovery process. ^53^ Generally, for CNS drugs, the physicochemical properties range from molecular weight 100 Da to 450 Da, hydrogen bond acceptors range from 0 to 5, hydrogen bond donor values range from 0 to 3, and the topological polar surface area value is ≤76 Å2. ^54^ The results of this study are listed in Table 2. The present study results are having lesser molecular weight and having similar hydrogen bond donor compared to previous ADME properties of 13 trial compounds with an IMID2 core scaffold molecular weight of 393.48, the hydrogen bond donor was 1. In the14 trial compound of the IMID2 core scaffold, the molecular weight was 451.56, and the hydrogen bond donors were 1. In the 15 trial compounds of the IMID2 core scaffold, the molecular weight was 336.39, and the hydrogen bond donors were 1. In 16 trials of the IMID2 core scaffold, the molecular weight was 394.47, and the number of hydrogen bond donors was 1. ^44^

**Table 2.** MM-GBSA score and Qikprop ADME prediction results of the top-9 selected compounds.

Name	MM-GBSA score	Molecular weight	Hydrogen Bond Donor	Hydrogen Bond Acceptor	Polar Surface Area
Z3336252116	-36.94	252.660	2	3	105.021
Z3136198649	-40.67	232.243	2	4	81.022
PV-005996498401	-33.07	232.243	2	3	101.470
PV-002589535286	-37.94	241.252	2	5.5	78.034
Z3603284828	-40.91	245.282	2	3	72.320
Z3136169169	-40.53	232.243	2	4	81.001
Z2497631540	-42.12	253.688	2	5.7	73.124
Z4468779454	-46.04	247.296	2	5.7	70.083
Z2708235883	-39.16	237.233	2	5.7	73.152

**The Z3336252116** with QPlogS value -1.653, QPlogPo/w value was 0.224, QPlogPw value was 14.263, QPlogBB value was -1.042, QPlogPC16 value was 8.612, QPlogPoct values was 15.179, QPPCaco values was 107.434, QPPMDCK values was 226.003. **Z3136198649** with QPlogS value -1.249, QPlogPo/w value was 0.180, QPlogPw value was 12.291, QPlogBB value was -0.286, QPlogPC16 value was 8.140, QPlogPoct values was 14.830, QPPCaco values was 155.541, QPPMDCK values was 73.234. **PV-005996498401** with QPlogS value -1.416, QPlogPo/w value was 0.223, QPlogPw value was 14.523, QPlogBB values with -1.051, QPlogPC16 value was 8.453, QPlogPoct values was 14.948, QPPCaco values was 159.830, QPPMDCK values was 143.876. **PV-0025895352867** with QPlogS value -2.886, QPlogPo/w value was 1.532, QPlogPw value was 11.558, QPlogBB value was -0.639, QPlogPC16 value was 9.016, QPlogPoct values was 14.584, QPPCaco values was 875.662, QPPMDCK values was 428.57. **Z3603284828** with QPlogS value -3.257, QPlogPo/w value was 2.117, QPlogPw value was 9.797, QPlogBB value was -0.530, QPlogPC16 value was 8.653, QPlogPoct values was 13.900, QPPCaco values was 1320.626, QPPMDCK values was 668.188. **Z3136169169** with QPlogS value -1.258, QPlogPo/w value was 0.181, QPlogPw value was 12.293, QPlogBB value was -0.290, QPlogPC16 value was 8.140, QPlogPoct values was 14.831, QPPCaco values was 155.076, QPPMDCK values was 72.997. **Z2497631540** with QPlogS value -3.149, QPlogPo/w value was 1.655, QPlogPw value was 10.508, QPlogBB value was -0.507, QPlogPC16 value was 8.503, QPlogPoct values was 14.075, QPPCaco values was 956.136, QPPMDCK values was 1160.160. **Z4468779454** with QPlogS value -2.896, QPlogPo/w value was 1.907, QPlogPw value was 10.321, QPlogBB value was -0.664, QPlogPC16 value was 8.871, QPlogPoct values was 14.100, QPPCaco values was 1311.747, QPPMDCK values was 663.334. **Z2708235883** with QPlogS value -2.771, QPlogPo/w value was 1.393, QPlogPw value was 10.536, QPlogBB value was -0.559, QPlogPC16 value was 7.537, QPlogPoct values was 13.626, QPPCaco values was 953.240, QPPMDCK values was 810.911.

Among all there parameter the QPlogBB is a useful parameter to predict or reflect directly the ability of a compound to cross the blood-brain barrier partition coefficient. ^55^ The recommended range of QPlogBB should be within -3.0 to 1.2. ^56^ If the compound is too polar then the compounds don’t cross the BBB. ^57^ The QPlogBB negative values indicate that the compounds are poorly permeable and polar. ^58^^,^^59^

### MDs analysis

Compounds were selected based on the screening process described above. Only short-listed compounds were subjected to desmonds for MDs. These studies provide information related to the stability and flexibility of the molecular docking complexes. Performed this study using the MM-GBSA. This study provides information related to protein-ligand interactions, RMSD variation can be assessed, and RMSF fluctuations can be assessed for protein-ligand complexes. The complex was regarded as stable if it fell within the 3 Å range. RMSD generally represents the average difference between the displacements of the atoms at an instant of target structures to the reference structure. As per MD, in comparison to the starting point, how the part of the structure changes or the entire structure over time or to identify large changes in protein structure.

RMSF is a calculation of a particular residue or individual residue fluctuation/flexibility or group of atoms during a simulation, related to the reference structure or average structure of simulation. ^60^ These are the compounds.

**Figure 2.**
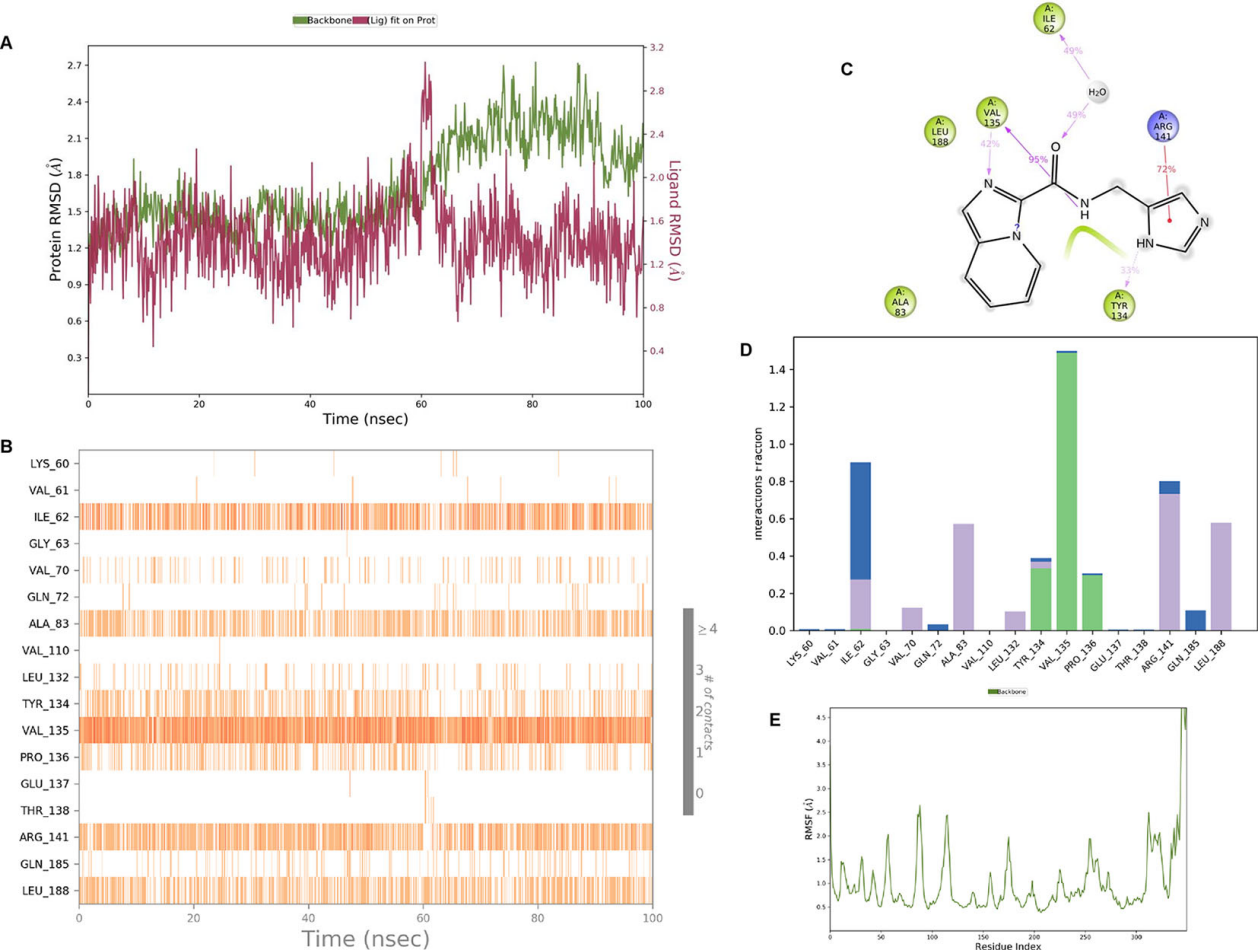
Pictorial representation of 6Y9R interactions with the IMID2 compound code PV-002589535286 monitored during MDs trajectory (A) Protein-Ligands RMSD; (B) Protein-Ligand Contacts; (C) Ligand-Protein Contacts; (D) Protein-Ligand Contacts described as histogram; (E) Protein RMSF.

**IMID2 PV-002589535286:** During MD simulations, the ligand-protein complex showed hydrogen-bonded interactions. The protein backbone and ligand structure exhibited higher RMSD fluctuations over the first 0-20 ns. The protein backbone and ligand fluctuations stayed within the range of 0.6 Å and 1.3 Å over the last 80 ns. The amino acid residue VAL135 formed a 95% direct hydrogen bonding interaction with the amide carbonyl, the amino acid residue VAL135 formed a 42% direct hydrogen bonding interaction with imidazole in the ring structure, the amino acid residue TYR134 had 33% direct hydrogen bonding interaction, and ARG141 had a 72% PI-cation interaction with 1H-imidazole. The amide carbonyl was exposed to an H _2_O molecule through which it interacted with residue ILE62 (49%), as shown in Figure 2; however, it was reported in **13** triads (previous literature) that the amide carbonyl was exposed to water-mediated contacts with residues such as ILE62 and GLN185 for the IMID2 core scaffold. The 16-triad isopropoxy group confirmed the probable water molecule interaction with the residue ASP200. The **17** triads showed an indirect interaction between the ortho substituent and residue ASN186. In the 18 triad, the di-F phenyl group was orientated towards LYS85, thus promoting an electrostatic interaction. However, VAL135 interaction formed direct hydrogen bonding interactions with amide carbonyl in all 13, 16, 17, and 18 trial compounds of previous IMID2 scaffolds. ^44^

Interaction diagram of 6Y9R with IMID2 compound code PV-002589535286 was observed during RMSD, Ligand interaction with amino acid residues of Protein Contact during MDs, protein-ligand contacts (cont.) (On the x-axis trajectory frame, the number is present, and amino acid residues are seen on the y-axis. The amino acid residues were in greater contact with ligands in the trajectory frame that appeared as a dark color shade). The VAL135 interaction fraction approached 1.4, whereas the ASP133 interaction fraction was missing in comparison to the core IMID2 scaffold from the previous literature. Protein root mean square fluctuation (RMSF) is mostly helpful for predicting changes that occur locally along the enzyme chain. These peaks provide information on how much the protein fluctuated during the MDs study.

**Figure 3.**
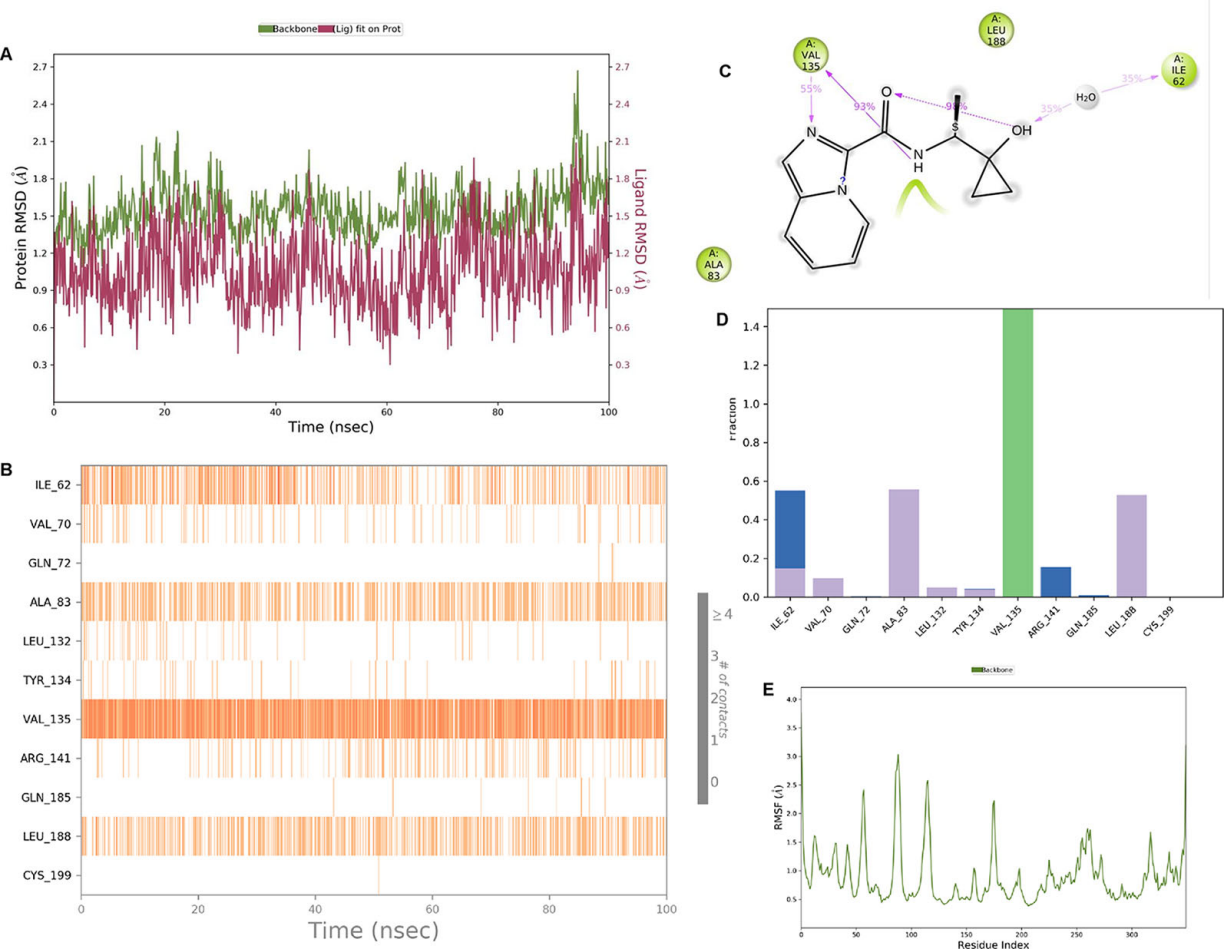
Pictorial representation of 6Y9R interaction with the IMID2 compound code Z3603284828 monitored during MDs trajectory (A) Protein-Ligands RMSD; (B) Protein-Ligand Contacts; (C) Ligand-Protein Contacts; (D) Protein-Ligand Contacts described as histogram; (E) Protein RMSF.

**IMID2 compound code Z3603284828:** During the MDs, the ligand-protein complex exhibited hydrogen-bonded interactions. The protein backbone and ligand structure exhibited higher RMSD fluctuations during the first 20 ns. The protein backbone and ligand fluctuations stayed within the range of 0.3 Å and 0.6 Å over the last 70 ns shown in Figure 3. The amino acid residue VAL135 formed 93% direct hydrogen bonding interaction with the amide carbonyl, the amino acid residue VAL135 formed 55% direct hydrogen bonding interaction with imidazole, and the amino acid residue ILE62 formed 35% water-mediated direct hydrogen bonding interaction with 1-hydroxycyclopropyl.

Interaction diagram of 6Y9R with IMID2 compound code Z3603284828 observed during RMSD, Ligand interaction with amino acid residues of Protein Contact during MDs, protein-ligand contacts (cont.) (On the x-axis, a trajectory frame with a number is present, and amino acid residues are seen on the y-axis. The amino acid residues that were in greater contact with ligands in the trajectory frame appeared as a dark color shade). The VAL135 interaction fraction approached 1.4, whereas the ASP133 interaction fraction was missing in comparison to the core IMID2 scaffold from previous literature, and protein RMSF is mostly helpful for predicting the changes that occur locally along the enzyme chain. These peaks provide information on how much the protein fluctuated during the MDs study.

**Figure 4.**
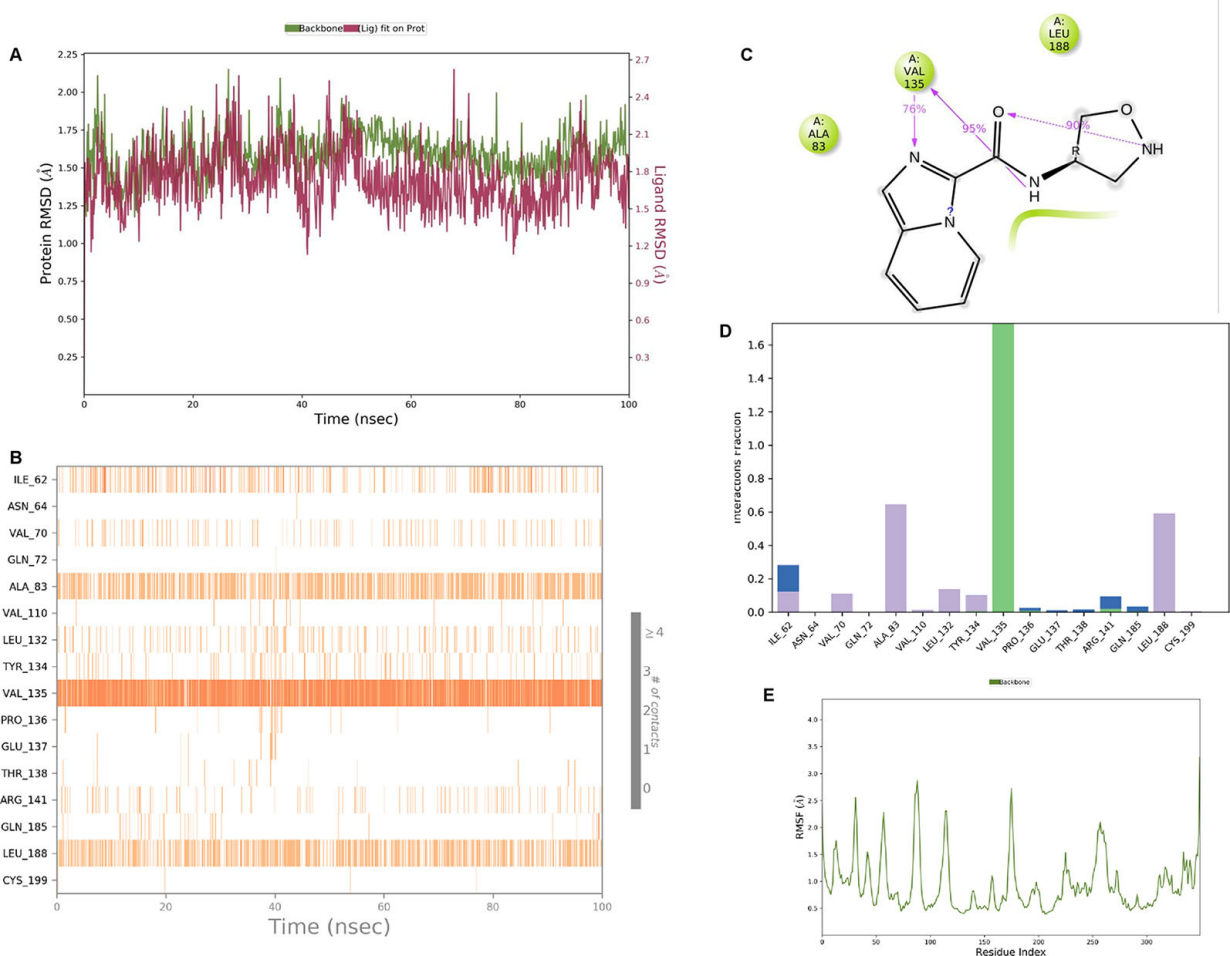
Pictorial representation of 6Y9R interaction with the IMID2 compound code Z3136169169 monitored during MDs trajectory (A) Protein-Ligand RMSD; (B) Protein-Ligands Contacts; (C) Ligand-Protein Contacts; (D) Protein-Ligand Contacts described as a histogram; (E) Protein RMSF.

**IMID2 compound code Z3136169169:** During the MDs, the ligand-protein complex exhibited hydrogen-bonded interactions. The protein backbone and ligand structure exhibited higher RMSD fluctuations during the first 20 ns. The protein backbone and ligand fluctuations stayed within the range of 0.8 Å and 0.3 Å over the last 70 ns shown in Figure 4. The amide carbonyl is exposed to hydrogen bonding and interacts with residue VAL135, and the amino acid residue VAL135 forms 76% direct hydrogen bonding interactions with imidazole.

Interaction diagram of 6Y9R with IMID2 compound code Z3136169169 observed during RMSD, Ligand interaction with amino acid residues of Protein Contact during MDs, protein-ligand contacts (cont.) (On the x-axis trajectory frame, the number is present, and amino acid residues are seen on the y-axis. The amino acid residues that were in greater contact with ligands in the trajectory frame appeared as a dark color shade). The VAL135 interaction fraction approached 1.6, whereas the ASP133 interaction fraction was missing in comparison to the core IMID2 scaffold from previous literature and protein RMSF is mostly helpful for predicting the changes that occur locally along with the enzyme chain. These peaks provide information on how much the protein fluctuated during the MDs study.

**Figure 5.**
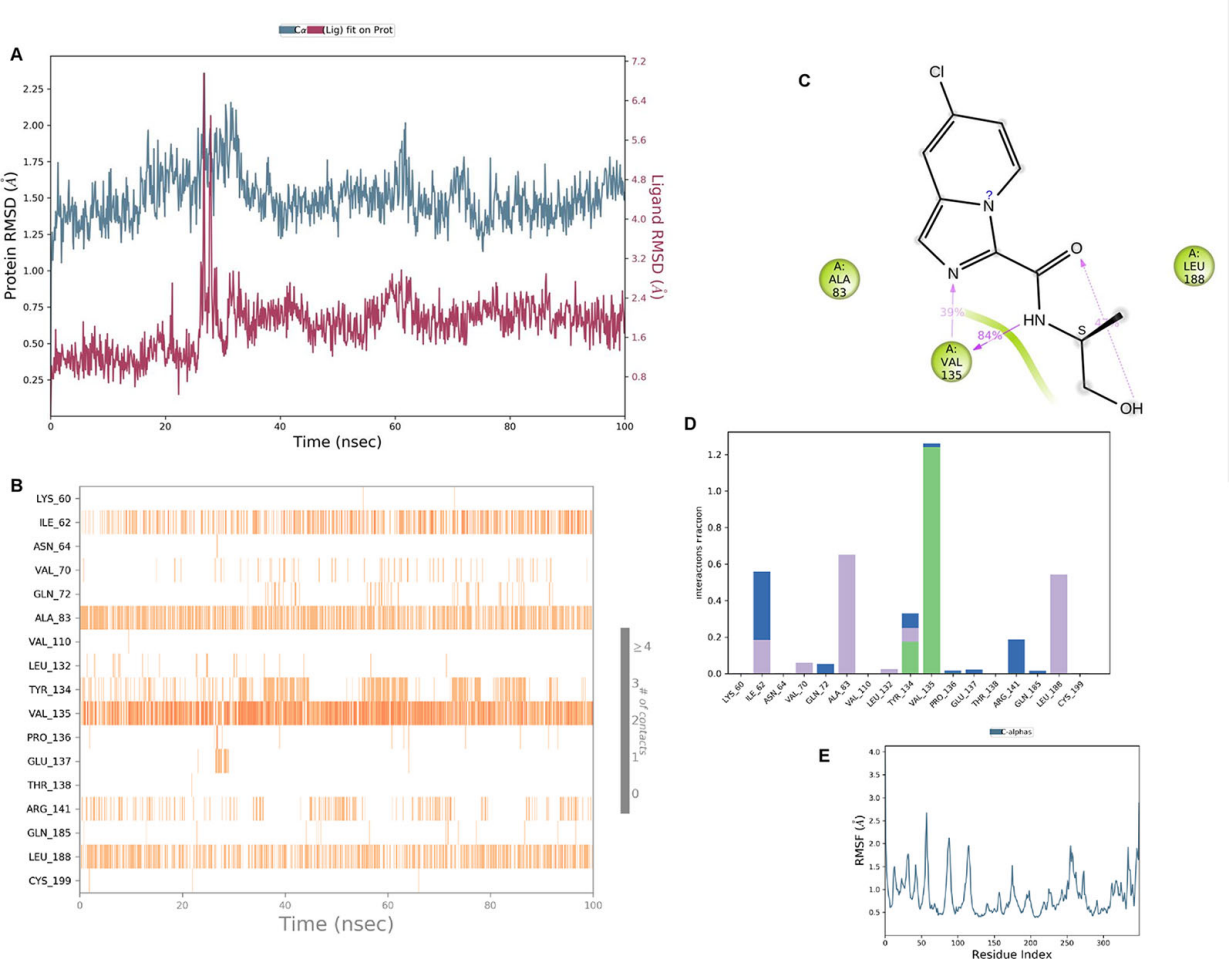
Pictorial representation of 6Y9R interaction with the IMID2 compound code Z2497631540 monitored during MDs trajectory (A) Protein-Ligand RMSD; (B) Protein-Ligand Contacts; (C) Ligand-Protein contacts; (D) Protein-Ligand Contacts described as a histogram; (E) Protein RMSF.

**IMID2 compound code Z2497631540:** During the MDs, the ligand-protein complex exhibited hydrogen-bonded interactions. The protein Cα and ligand structures exhibited higher RMSD fluctuations over the first 20 ns. The protein backbone and ligand fluctuations stayed within the range of 0.4 Å and 3.7 Å over the last 70 ns shown in Figure 5. The amino acid residue VAL135 formed 84% direct hydrogen bonding interactions with the amide carbonyl, and the amino acid residue VAL135 formed 39% direct hydrogen bonding interactions with imidazole in the ring structure.

Interaction diagram of 6Y9R with IMID2 compound code Z2497631540 observed during RMSD, Ligand interaction with amino acid residues of Protein Contact during MDs, protein-ligand contacts (cont.) (On the x-axis, a trajectory frame with a number is present, and amino acid residues are seen on the y-axis. The amino acid residues that were in greater contact with ligands in the trajectory frame appeared as a dark color shade). The VAL135 interaction fraction approached 1.2, whereas the ASP133 interaction fraction was missing in comparison to the core IMID2 scaffold from previous literature, and protein RMSF is mostly helpful for predicting the changes that occur locally along the enzyme chain. These peaks provide information on how much the protein fluctuated during the MDs study.

**Figure 6.**
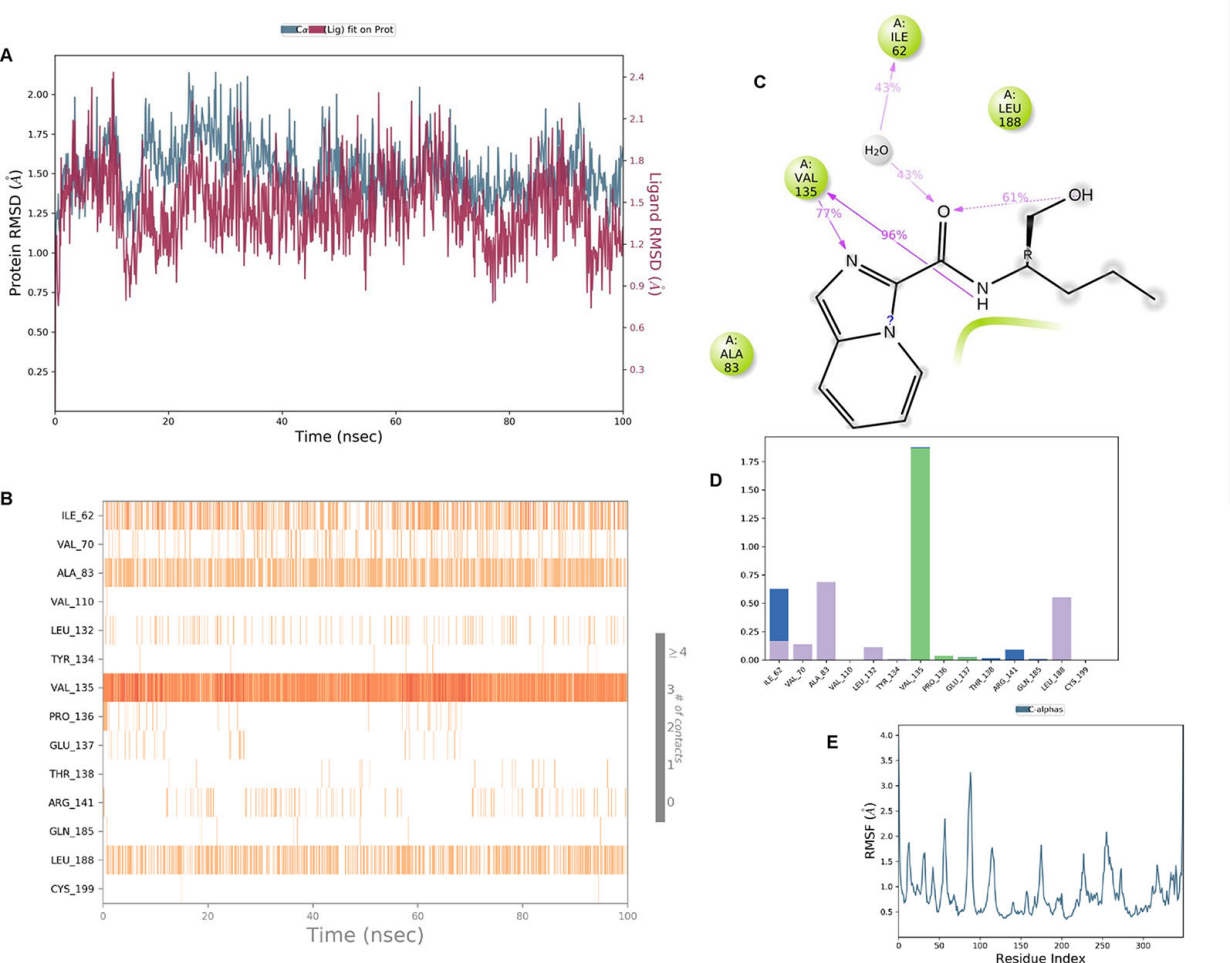
Pictorial representation of 6Y9R interaction with the IMID2 compound code Z4468779454 monitored during MDs trajectory (A) Protein-Ligand RMSD; (B) Protein-Ligand contacts; (C) Ligand-Protein contacts; (D) Protein-Ligand contacts described as a histogram; (E) Protein RMSF.

**IMID2 compound code Z4468779454:** During the MDs, the ligand-protein complex exhibited hydrogen-bonded interactions. The protein Cα and ligand structures exhibited higher RMSD fluctuations over the first 20 ns. The protein backbone and ligand fluctuations stayed within the range of 0.5 Å and 0.4 Å over the last 70 ns shown in Figure 6. The amino acid residue VAL135 formed a 96% direct hydrogen bonding interaction with the amide carbonyl, the amino acid residue VAL135 formed a 77% direct hydrogen bonding interaction with imidazole, and the amide carbonyl was exposed to the H _2_O molecule through which it interacted with residue ILE62, with 43% and 61% interaction with 1-hydroxypentan-2-yl.

Interaction diagram of 6Y9R with IMID2 compound code Z4468779454 observed during RMSD, Ligand interaction with amino acid residues of Protein Contact during MDs, protein-ligand contacts (cont.) (On the x-axis trajectory frame, the number is present, and amino acid residues are seen on the y-axis. The amino acid residues were in greater contact with ligands in the trajectory frame that appeared as a dark color shade). The VAL135 interaction fraction approached 1.75, whereas the ASP133 interaction fraction was missing in comparison to the core IMID2 scaffold from previous literature, and protein RMSF is mostly helpful for predicting the changes that occur locally along the enzyme chain. These peaks provide information on how much the protein fluctuated during the MDs study.

**Figure 7.**
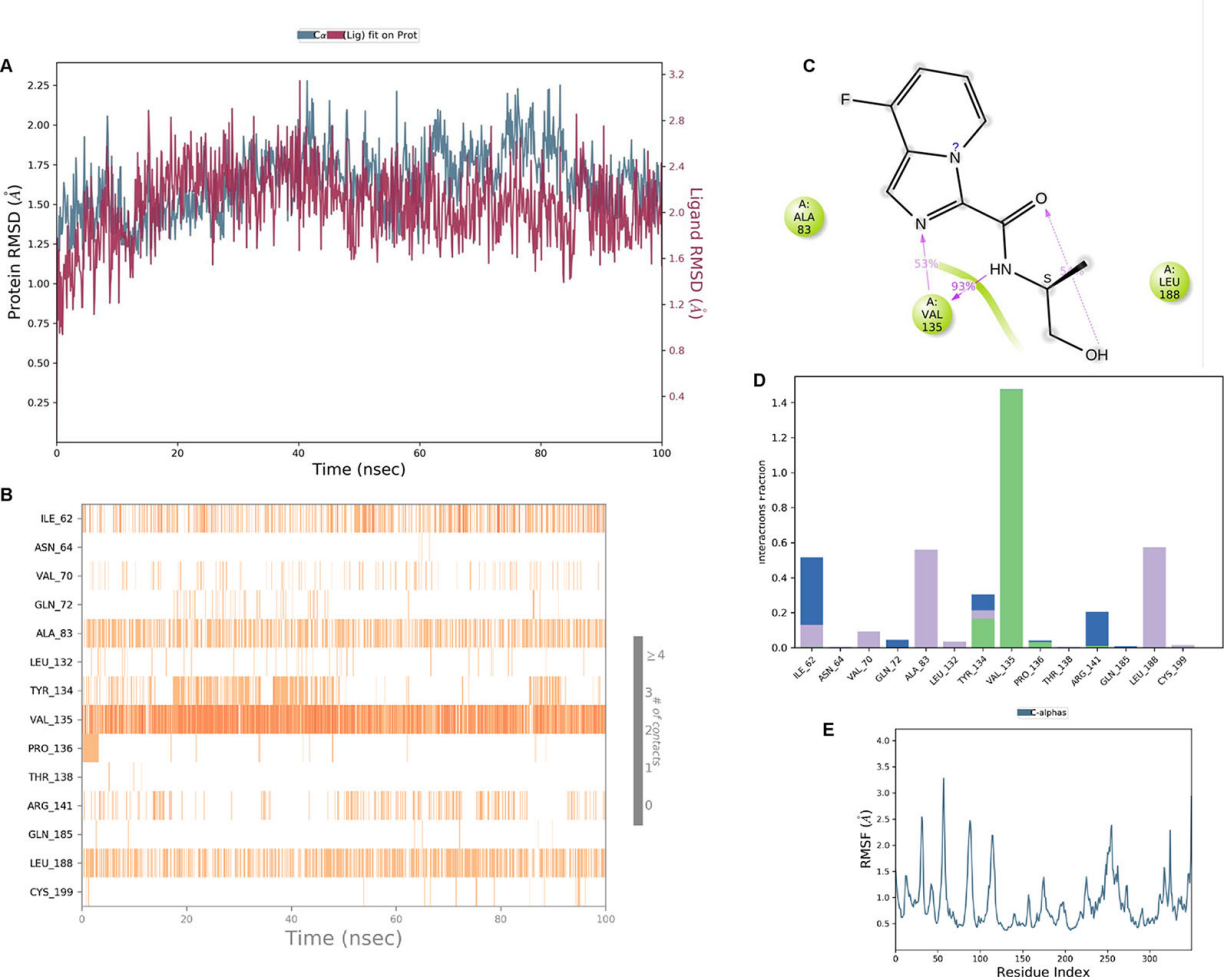
Pictorial representation of 6Y9R interaction with the IMID2 compound code Z2708235883 monitored during MDs trajectory (A) Protein-Ligand RMSD; (B) Protein-Ligand Contacts; (C) Ligand-Protein Contacts; (D) Protein-Ligand Contacts described as a histogram; (E) Protein RMSF.

**IMID2 compound code Z2708235883:** During the MDs, the ligand-protein complex exhibited hydrogen-bonded interactions. The protein backbone and ligand structure exhibited higher RMSD fluctuations during the first 20 ns. The protein Cα and ligand fluctuations stayed within the range of 0.5 Å and 0.2 Å over the last 70 ns. The amino acid residue VAL135 formed 93% direct hydrogen bonding interactions with the amide carbonyl, and the amino acid residue VAL135 formed 53% direct hydrogen bonding interactions with imidazole in the ring structure, as shown in Figure 7.

Interaction diagram of 6Y9R with IMID2 compound code Z2708235883 observed during RMSD, Ligand interaction with amino acid residues of Protein Contact during MDs, protein-ligand contacts (cont.) (On the x-axis trajectory frame, the number is present, and amino acid residues are seen on the y-axis. The amino acid residues that have been in more contact with ligands in the trajectory frame appear to have a dark color shade). The VAL135 interaction fraction approached 1.4, whereas the ASP133 interaction fraction was missing in comparison to the core IMID2 scaffold from previous literature, and protein RMSF is mostly helpful for predicting the changes that occur locally along the enzyme chain. These peaks provide information on how much the protein fluctuated during the MDs study.

**Figure 8.**
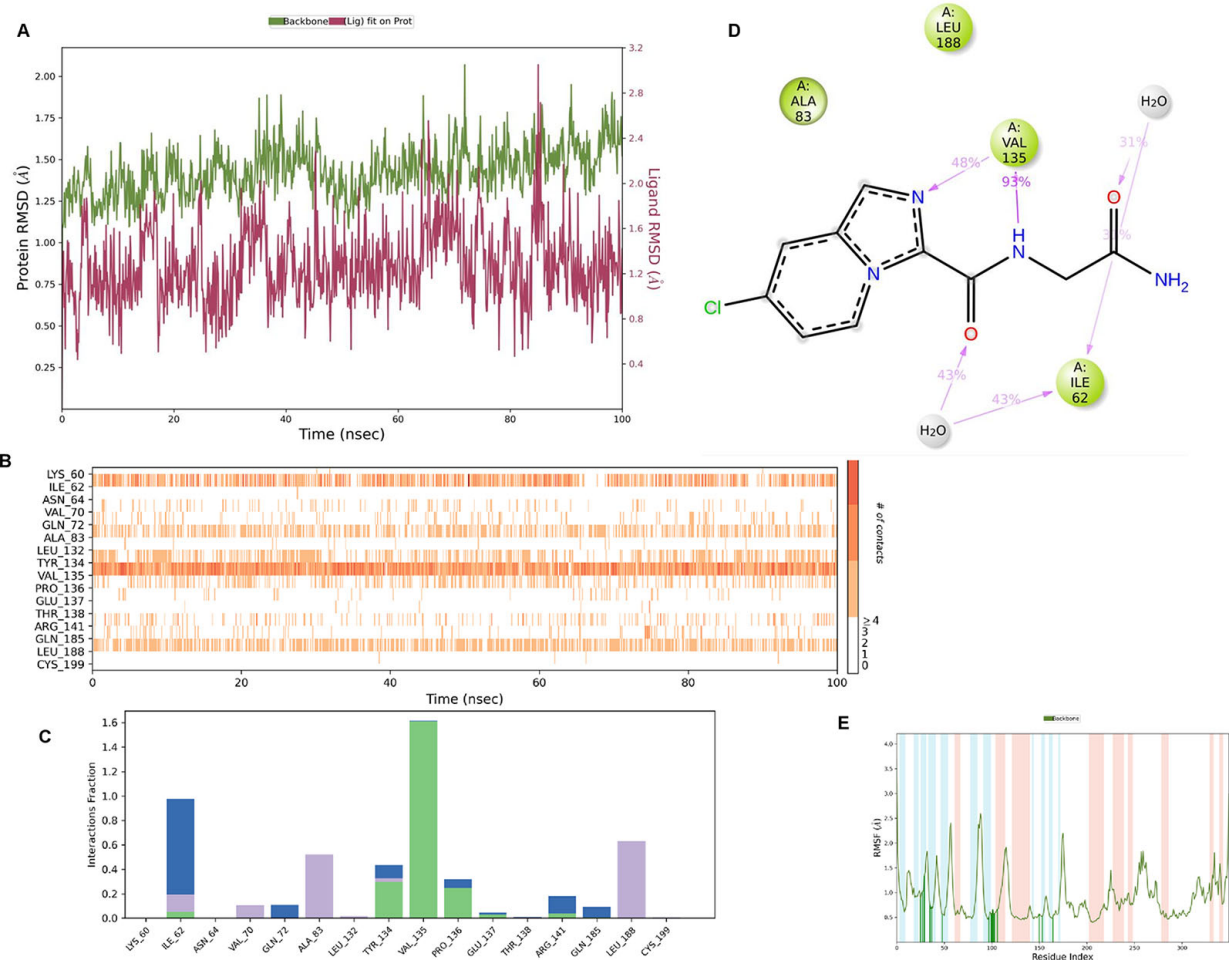
Pictorial representation of 6Y9R interaction with the IMID2 compound code Z3336252116 monitored during MDs trajectory (A) Protein-Ligand RMSD; (B) Protein-Ligand Contacts; (C) Protein-Ligand Contacts described as a histogram; (D) Ligand-Protein Contacts; (E) Protein RMSF.

**IMID2_compound code Z3336252116**: During MD simulations, the ligand-protein complex showed hydrogen-bonded interactions. The protein backbone and ligand structure exhibited higher RMSD fluctuations over the first 0-20 ns. The protein backbone and ligand fluctuations stayed within the range of 0.6 Å and 2.2 Å over the last 80 ns. The amino acid residue VAL135 formed 93% direct hydrogen bonding interactions with the amide carbonyl, and the amino acid residue VAL135 formed 48% direct hydrogen bonding interactions with imidazole in the ring structure, which was exposed to the H _2_O molecule through which it interacted with residue ILE62 (43%), as shown in Figure 8.

Interaction diagram of 6Y9R with IMID2_compound code Z3336252116 observed during RMSD, Ligand interaction with amino acid residues of Protein Contact during MDs, protein-ligand contacts (cont.) (On the x-axis trajectory frame, the number is present, and amino acid residues are seen on the y-axis. The amino acid residues that have been in more contact with ligands in the trajectory frame appear to have a dark color shade). The VAL135 interaction fraction approached 1.5, whereas the ASP133 interaction fraction was missing in comparison to the core IMID2 scaffold from the previous literature. Protein RMSF is mostly helpful for predicting changes that occur locally along the enzyme chain. These peaks provide information on how much the protein fluctuated during MDs study.

**Figure 9.**
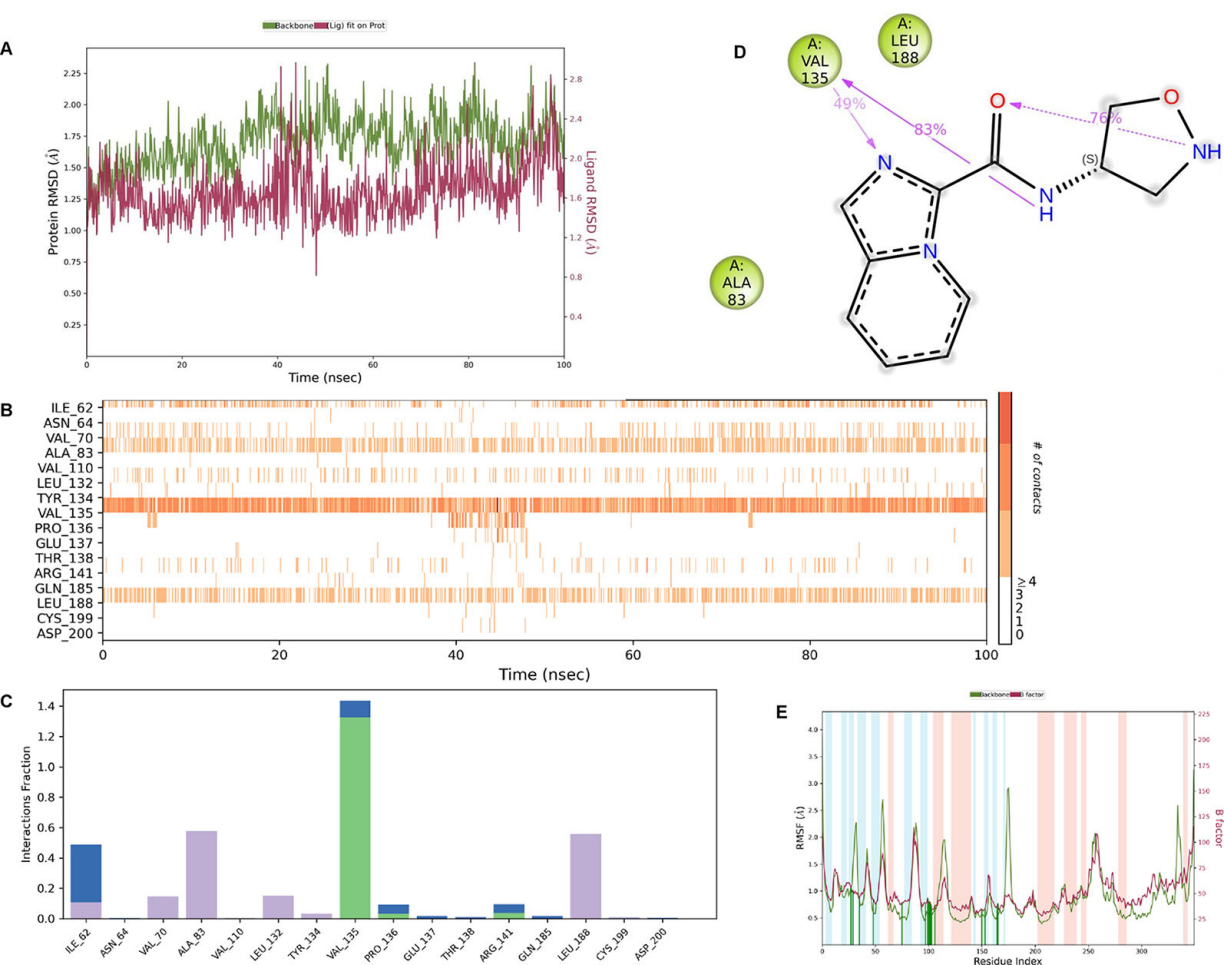
Pictorial representation of 6Y9R interactions with the IMID2 compound code Z3136198649 monitored during MDs trajectory (A) Protein-Ligands RMSD; (B) Protein-Ligand Contacts; (C) Protein-Ligand Contacts described as a histogram; (D) Ligand-Protein Contacts; (E) Protein RMSF.

**IMID2 compound code Z3136198649**: During MDs, the ligand-protein complex showed hydrogen-bonded interactions. The protein backbone and ligand structure exhibited higher RMSD fluctuations over the first 0-20 ns. The protein backbone and ligand fluctuations stayed within the range of 0.7 Å and 0.5 Å over the last 80 ns. The amino acid residue VAL135 formed 83% direct hydrogen bonding interaction with amide carbonyl, and the amino acid residue VAL135 formed 49% direct hydrogen bonding interaction with imidazole in the ring structure given in Figure 9.

Interaction diagram of 6Y9R with IMID2 compound code Z3136198649 observed during RMSD, Ligand interaction with amino acid residues of Protein Contact during MDs, protein-ligand contacts (cont.) (On the x-axis trajectory frame, the number is present, and amino acid residues are seen on the y-axis. The amino acid residues that have been in more contact with ligands in the trajectory frame appear to have a dark color shade). The VAL135 interaction fraction approached 1.4, whereas the ASP133 interaction fraction was missing in comparison to the core IMID2 scaffold from the previous literature. Protein RMSF is mostly helpful for predicting changes that occur locally along the enzyme chain. These peaks provide information on how much the protein fluctuated during the MDs study.

**Figure 10.**
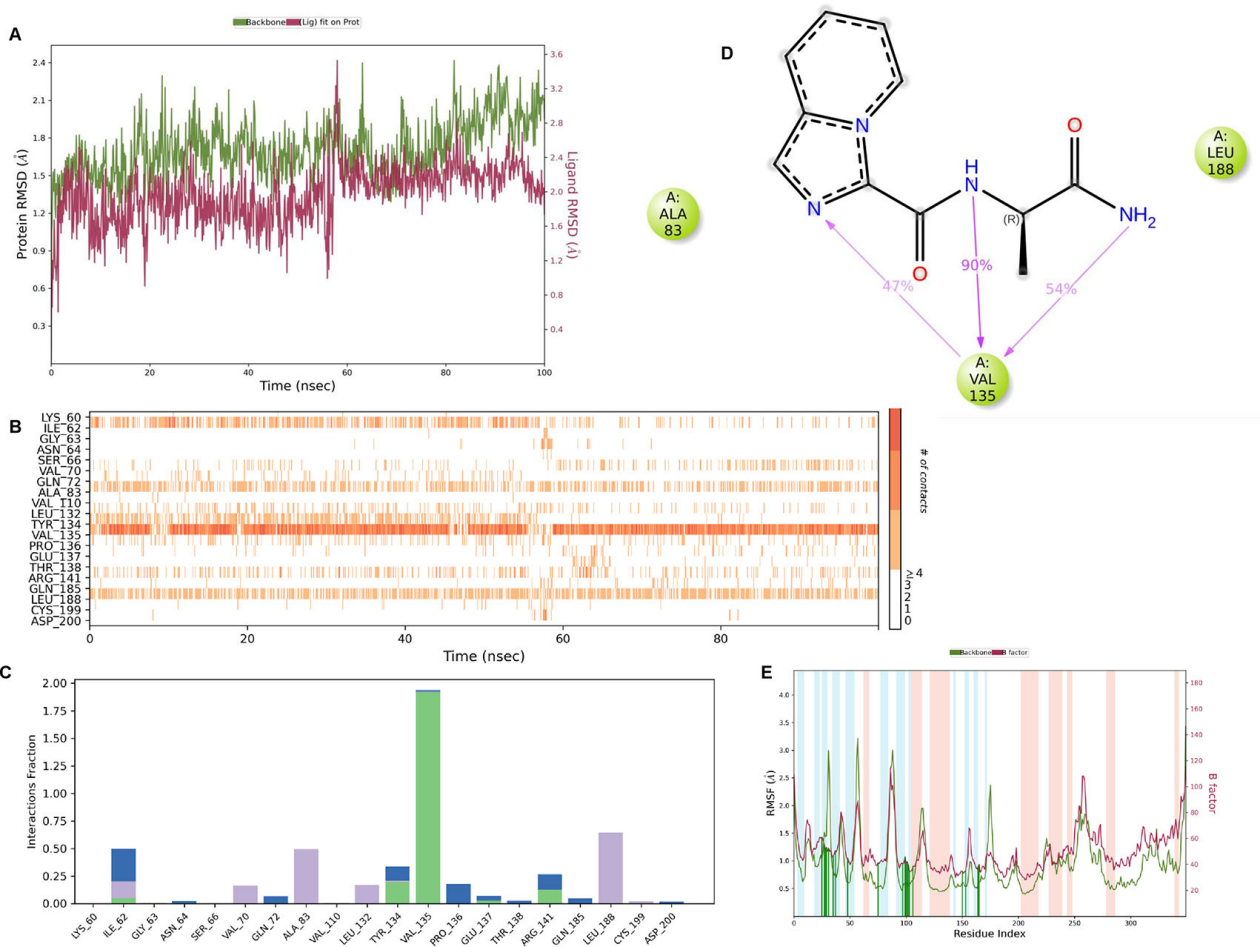
Pictorial representation of 6Y9R interaction with the IMID2 compound code PV-005996498401 monitored during MDs trajectory (A) Protein-Ligand RMSD; (B) Protein-Ligand contacts; (C) Protein-Ligand contacts described as a histogram; (D) Ligand-Protein contacts; (E) Protein RMSF.

**IMID2 compound code PV-005996498401**: During MDs, the ligand-protein complex showed hydrogen-bonded interactions. The protein backbone and ligand structure exhibited higher RMSD fluctuations over the first 0-20 ns. The protein backbone and ligand fluctuations stayed within the range of 1.0 Å and 0.6 Å over the last 80 ns. The amino acid residue VAL135 formed 90% direct hydrogen bonding interactions with the amide carbonyl, and the amino acid residue VAL135 formed 47% direct hydrogen bonding interactions with imidazole in the ring structure, as shown in Figure 10.

Interaction diagram of 6Y9R withIMID2 compound code PV-005996498401 observed during RMSD, Ligand interaction with amino acid residues of Protein Contact during MDs, protein-ligand contacts (cont.) (On the x-axis trajectory frame, the number is present, and amino acid residues are seen on the y-axis. The amino acid residues that have been in more contact with ligands in the trajectory frame appear to have a dark color shade). The VAL135 interaction fraction approached 1.85, whereas the ASP133 interaction fraction was missing in comparison to the core IMID2 scaffold from the previous literature. Protein RMSF is mostly helpful for predicting changes that occur locally along the enzyme chain. These peaks provide information on how much the protein fluctuated during the MDs study.

## Conclusion

In this study, the focused library generation was selected to target the receptor grid region (ATP-competitive site) of 6Y9R. After docking, the compounds were separated and selected based on the VAL135 residue interaction. Further prediction was performed using Qikprop and Prime MM-GBSA assays and MDs studies. Among all the MDs studies the compound (Z3336252116) has shown good interaction and good docking score. Further experimental studies are required to confirm these findings.

### Ethical considerations

Ethics and written consent were not applicable.

## Data Availability

Figshare: Molecular docking studies and molecular dynamic simulation to identify GSK-3β inhibitors for AD, https://doi.org/10.6084/m9.figshare.24592716.v1. ^61^ The underlying data for this project are:
➢
Enamine_IMID2_Scaffold.csv ➢
Enamine_IMID2_Scaffold_with nitrogen_realdb_molecules 17_35_52.sdf ➢
Ligprep_enamine_IMID2_Scaffold_withnitrogen_realdb_out.csv ➢
Ligprep_enamine_IMID2_SCAFFOLD_WITHNITROGEN_REALDB-out.mae ➢
Prime_mmgbsa_3_out.csv ➢
prime_mmgbsa_3-out.maegz ➢
Prime_mmgbsa_VAL135_INTERACTION_IMID2_ENAMINE_WITHNITROGEN-out.csv ➢
prime_mmgbsa_VAL135_INTERACTION_IMID2_ENAMINE_WITHNITROGEN-out.maegz ➢
QIKPROP_LIGPRE_IMID2_ENAMINE_WITHNITROGEN-out.csv ➢
QIKPROP_LIGPREP_ENAMINE_IMID2_SCAFFOLD_WITHNITROGEN_REALDB-out.mae Data are available under the terms of the Creative Commons Attribution 4.0 International license (CC-BY 4.0). Protein Data Bank: 6Y9R, https://doi.org/10.2210/pdb6Y9R/pdb. ^62^
