# Supplementary material for: Molecular docking studies and molecular dynamic simulation analysis: To identify novel ATP-competitive inhibition of Glycogen synthase kinase-3β for Alzheimer’s disease
Source: F1000Res. 2025 May 27;13:773. Originally published 2024 Jul 8. [Version 3] doi: 10.12688/f1000research.145391.3 (PMC12121352; doi:10.12688/f1000research.145391.3)
Supplement: Peer review history

# Reviewer report: Reviewer response for version 2

**DOI:** 10.5256/f1000research.170347.r318530

**Published:** 2024-09-28

**Authors:** Sachchida Rai, Payal Singh

**Affiliations:** 1 Centre of Experimental Medicine and Surgery (CEMS), Institute of Medical Sciences (IMS), Banaras Hindu University, Varanasi, Uttar Pradesh, 221005, India; 2 Department of Zoology, Banaras Hindu University, Varanasi, Uttar Pradesh, India

**I have some questions on the manuscript which are as follows.**

 How do the identified GSK-3β inhibitors compare to existing therapeutics in terms of binding affinity and specificity?

 What specific modifications were made to the previously used computational methods, and how did they enhance the prediction accuracy?

 Can you provide more details on the ligand candidates identified? Were any novel chemical structures or scaffolds discovered?

 How do the ADME predictions of the identified ligands suggest their potential success in in vivo studies?

 Were there any challenges encountered in using the computational simulation tools, and how were they addressed in this study?

 Have you considered testing the identified GSK-3β inhibitors in relevant Alzheimer’s disease animal models?

 Did the molecular dynamics simulations provide insights into the stability and conformational changes of the protein-ligand complexes over time?

 How do you plan to validate the computational results experimentally, and what are the next steps in your drug development pipeline?

 Was there any off-target effects observed for the identified inhibitors in the computational analyses?

 Include some relevant bibliographic studies like Ramakrishna K, et al., 2024 (Ref 1), Tripathi PN, et al., 2024 (Ref 2), Singh M, et al., 2024 (Ref 3), Tripathi PN, et al., 2019 (Ref 4), Srivastava P, et al., 2019 (Ref 5), & Rai SN, et al., 2020 (Ref 6) in your manuscript.

 Could targeting GSK-3β alone be sufficient to address the complex pathology of Alzheimer's disease, or do you propose a combination therapy approach?

 How did the focused library generation improve the targeting accuracy of the ATP-competitive site in 6Y9R?

 What criteria were used to select compounds based on the VAL135 residue interaction, and how critical is this interaction for inhibitory activity?

 How reliable are the Qikprop and Prime MM-GBSA assays in predicting the efficacy of the compounds before experimental validation?

 Can you elaborate on the molecular dynamics simulation results, specifically regarding the stability of the protein-ligand complexes?

 Were any alternative residues besides VAL135 considered for interaction, and how might they impact the binding efficiency?

 How were the docking scores correlated with the Qikprop predictions and MM-GBSA binding energy calculations?

 What experimental techniques do you propose to validate the computational findings, and what challenges do you anticipate in this process?

 How do the identified compounds compare to existing inhibitors of 6Y9R in terms of predicted binding affinity and selectivity?

 Did the molecular dynamics simulations reveal any significant conformational changes in the receptor that might affect ligand binding?

 How do you plan to prioritize the compounds for further experimental testing, given the computational predictions?

Is the work clearly and accurately presented and does it cite the current literature?

Yes

If applicable, is the statistical analysis and its interpretation appropriate?

Yes

Are all the source data underlying the results available to ensure full reproducibility?

Yes

Is the study design appropriate and is the work technically sound?

Yes

Are the conclusions drawn adequately supported by the results?

Yes

Are sufficient details of methods and analysis provided to allow replication by others?

Yes

Reviewer Expertise:

My research focuses on exploring the molecular mechanisms and therapeutic interventions for neurodegenerative diseases, particularly Alzheimer's and Parkinson's disease. I investigate the roles of biomarkers, signaling pathways, and epigenetic regulators in disease progression. My work integrates computational biology, drug discovery, and experimental models to identify novel therapeutic targets, including GSK-3β and other key enzymes. I also explore natural compounds, such as those derived from nutraceuticals and endophytic fungi, for their neuroprotective potential. Additionally, I study autophagy, oxidative stress, and inflammation as critical factors in neurodegeneration, aiming to develop targeted strategies for chronic disease mitigation and brain health.

We confirm that we have read this submission and believe that we have an appropriate level of expertise to confirm that it is of an acceptable scientific standard, however we have significant reservations, as outlined above.

## References

[1] : Indole-3 Carbinol and Diindolylmethane Mitigated β-Amyloid-Induced Neurotoxicity and Acetylcholinesterase Enzyme Activity: In Silico, In Vitro, and Network Pharmacology Study. *Diseases* .2024;12(8) : 10.3390/diseases12080184 10.3390/diseases12080184 39195183 PMC11354007

[2] : Review of Pharmacotherapeutic Targets in Alzheimer's Disease and Its Management Using Traditional Medicinal Plants. *Degener Neurol Neuromuscul Dis* .2024;14: 10.2147/DNND.S452009 47-74 10.2147/DNND.S452009 38784601 PMC11114142

[3] : A Comprehensive Review and Androgen Deprivation Therapy and Its Impact on Alzheimer's Disease Risk in Older Men with Prostate Cancer. *Degener Neurol Neuromuscul Dis* .2024;14: 10.2147/DNND.S445130 33-46 10.2147/DNND.S445130 38774717 PMC11108066

[4] : Biphenyl-3-oxo-1,2,4-triazine linked piperazine derivatives as potential cholinesterase inhibitors with anti-oxidant property to improve the learning and memory. *Bioorg Chem* .2019;85: 10.1016/j.bioorg.2018.12.017 82-96 10.1016/j.bioorg.2018.12.017 30605887

[5] : Design and development of some phenyl benzoxazole derivatives as a potent acetylcholinesterase inhibitor with antioxidant property to enhance learning and memory. *Eur J Med Chem* .2019;163: 10.1016/j.ejmech.2018.11.049 116-135 10.1016/j.ejmech.2018.11.049 30503937

[6] : Mitochondrial Dysfunction: a Potential Therapeutic Target to Treat Alzheimer's Disease. *Mol Neurobiol* .2020;57(7) : 10.1007/s12035-020-01945-y 3075-3088 10.1007/s12035-020-01945-y 32462551

# Reviewer report: Reviewer response for version 1

**DOI:** 10.5256/f1000research.159332.r302514

**Published:** 2024-10-10

**Authors:** Shvetank Bhatt

**Affiliations:** 1 Dr. Vishwanath Karad MIT World Peace University, Pune, Maharashtra, India

The manuscript is well written and can be accepted after minor modifications.

 1. Author can highlight the names of drugs which are recently approved for the treatment of AD in introduction section.

 2. Results and discussion section is presented well by authors.

 3. Author should discuss the epidemiology and  pathophysiology of diseases briefly to understand the severity of disease.

Is the work clearly and accurately presented and does it cite the current literature?

Yes

If applicable, is the statistical analysis and its interpretation appropriate?

I cannot comment. A qualified statistician is required.

Are all the source data underlying the results available to ensure full reproducibility?

Yes

Is the study design appropriate and is the work technically sound?

Yes

Are the conclusions drawn adequately supported by the results?

Yes

Are sufficient details of methods and analysis provided to allow replication by others?

Yes

Reviewer Expertise:

CNS Diosrders, AD, Depression,  Anxiety

I confirm that I have read this submission and believe that I have an appropriate level of expertise to confirm that it is of an acceptable scientific standard.

# Reviewer report: Reviewer response for version 1

**DOI:** 10.5256/f1000research.159332.r302510

**Published:** 2024-09-13

**Authors:** Jigna Samir Shah

**Affiliations:** 1 Nirma University, Ahmedabad, Gujarat, India

1. Abstract can be modified. Also, in conclusion no specific compound was identified.

 2. Role of GSK-3β in pathogenesis of AD can be explored more. Mechanism of GSK-3β in AD can be defined briefly in introduction section.

 3. Why this PDB ID 6Y9R was selected from 89 IDs as mentioned in results and

 discussion. It can be elaborated briefly. Also, besides this domain, GSK-3β inhibition might be possible through different binding sites. If possible, it can also be explored.

 4. There are grammatical errors and improper sentence formations in manuscript, which can be modified. Also, instead of using “we”, the authors can used indirect speech.

 5. Full forms mentioned are not consistent. Mention the full form in first use and thereafter abbreviations can be used.

 6. In result sections, “Protein root mean square fluctuation (RMSF) is mostly helpful for predicting changes that occur locally along the enzyme chain.” is repeatedly mentioned in each result. The authors can mention the significance of findings like RMSD, RMSF at the start in a separate paragraph, or only once in initial results. Thereafter it can be understood.

 7. In ADME studies, it is mentioned that QPlogBB (predicted brain/blood partition coefficient), was predicted however, in results it is not mentioned. It can be mentioned, to evaluate blood brain barrier permeability. In AD, since the target region for therapy is brain, BBB permeability is a significant parameter.

 8. No standard drug or inhibitor is included in MDS studies. If possible, a standard can be taken into consideration.

 9. There is no discussion about findings of the article, and conclusion is very vague. At the end of study, there is no comparison of the 9 compounds screened. Better compound should be identified and screened. Biological data to support the findings can be added, however it is not mandatory. Discussion section should be added and findings of each compound should be compared and correlated to previously done studies.

 10. Discuss future prospects of this study, elaborate briefly what experimental studies should be done further and state the limitations of this study.

Is the work clearly and accurately presented and does it cite the current literature?

Yes

If applicable, is the statistical analysis and its interpretation appropriate?

Not applicable

Are all the source data underlying the results available to ensure full reproducibility?

Yes

Is the study design appropriate and is the work technically sound?

Yes

Are the conclusions drawn adequately supported by the results?

Yes

Are sufficient details of methods and analysis provided to allow replication by others?

Yes

Reviewer Expertise:

Neurodegenerative diseases, oral cancer, breast cancer

I confirm that I have read this submission and believe that I have an appropriate level of expertise to confirm that it is of an acceptable scientific standard.

# Reviewer report: Reviewer response for version 1

**DOI:** 10.5256/f1000research.159332.r302509

**Published:** 2024-07-29

**Authors:** Qingchun Zhao

**Affiliations:** 1 Shenyang Pharmaceutical University, Shenyang, China

In this paper, the authors used computer simulation technology to select 9 compounds with GSK3 as the target, and then studied ADME prediction and molecular docking through different modules.

 Keep reading the paper, three questions are raised.

 1. In the abstract, the author describes the method as "we have used different modules that were used in previous studies with a little modification", but in fact, the method used by the author is a routine application, and there is no prominent modification.

 2. In the introduction section, is there only one sentence to summarize the article?

 3.The content of the whole study is simple, the innovation is poor, and the workload is less, which is not enough to support a research article.

 Therefore, it is hoped that the author can modify some contents of the article and add some follow-up experiments to make the article more comprehensive.

Is the work clearly and accurately presented and does it cite the current literature?

Yes

If applicable, is the statistical analysis and its interpretation appropriate?

Yes

Are all the source data underlying the results available to ensure full reproducibility?

Yes

Is the study design appropriate and is the work technically sound?

Partly

Are the conclusions drawn adequately supported by the results?

Partly

Are sufficient details of methods and analysis provided to allow replication by others?

Yes

Reviewer Expertise:

Alzheimer's Disease, Cancer , Natural Pharmaceutical Chemistry, Pharmaceutical chemistry

I confirm that I have read this submission and believe that I have an appropriate level of expertise to state that I do not consider it to be of an acceptable scientific standard, for reasons outlined above.

# Response

**Published:** 2024-08-13

**Authors:** Sreedhara Ranganath Pai

**Affiliations:** Manipal college of pharmaceutical sciences, India

**Reviewer Comment 1:** In the abstract, the author describes the method as "we have used different modules that were used in previous studies with a little modification", but in fact, the method used by the author is a routine application, and there is no prominent modification.

**Response to comment 1**: We used Maestro, a graphical interface of Schrodinger, for our computational simulation studies. In the present work, we have used different modules such as Protein Preparation Wizard for Protein Preparation, LigPrep for Ligand Preparation, Qikprop for ADME (Absorption, Distribution, Metabolism and Excretion) prediction, Glide for docking studies, Prime for Binding energy prediction and Desmond for Molecular dynamic simulation studies used.

**Reviewer Comment 2**: In the introduction section, is there only one sentence to summarize the article?

**Response to comment 2**: For better BBB permeation, the structure was finalized based on a wet lab. So, based on this literature search, we have selected core imidazole scaffold for our study, and from that core imidazole scaffold, we have drawn sub-structures in the enamine database. Then docking studies were done on the compounds and subjected to ADME (Qikprop). A molecular dynamic simulation study was conducted on nine compounds.

**Reviewer Comment 3**: The content of the whole study is simple, the innovation is poor, and the workload is less, which is not enough to support a research article.

 Therefore, it is hoped that the author can modify some contents of the article and add some follow-up experiments to make the article more comprehensive.

**Response to comment 3**: The reviewer's comment is completely one-sided. We regret to answer this comment, and we completely disagree with the reviewer's suggestion, as this work took 18 months from the conceptualization of the idea. This work is computational modelling, and our objectives are clear, and in our view, the data generated is sufficient to make the conclusion.
